# The fibrin-derived peptide FX06 protects human pulmonary endothelial cells against the COVID-19-triggered cytokine storm

**DOI:** 10.3389/fimmu.2025.1591860

**Published:** 2025-06-19

**Authors:** Zhiran Wang, Dmitrii Lebedev, Simeng Li, Sudharshan Rao, Kevin Wu, Lorcan Doyle, Kieran Wynne, Alfonso Blanco, Margaritha M. Mysior, Jeremy C. Simpson, Dimitri Scholz, Petra Wülfroth, Kai Zacharowski, Walter Kolch, Vadim Zhernovkov, Günther Eissner

**Affiliations:** ^1^ Systems Biology Ireland, School of Medicine, University College Dublin, Dublin, Ireland; ^2^ Flow Cytometry Core, Conway Institute of Biomolecular and Biomedical Research, University College Dublin, Dublin, Ireland; ^3^ Cell Screening Laboratory, School of Biology and Environmental Science, O’Brien Centre for Science, University College Dublin, Dublin, Ireland; ^4^ Imaging Core, Conway Institute of Biomolecular and Biomedical Research, University College, Dublin, Ireland; ^5^ F4 Pharma, Vienna, Austria; ^6^ Clinic of Anesthesiology, Intensive Care Medicine and Pain Therapy, University Hospital Frankfurt, Frankfurt, Germany

**Keywords:** endothelial dysfunction, COVID-19, cytokine, FX06, vascular leakage, ARDS

## Abstract

**Introduction:**

Coronavirus disease 2019 (COVID-19), caused by severe acute respiratory syndrome coronavirus 2 (SARS-CoV-2), has been a major health emergency since its emergence in late 2019. Endothelial dysfunction is a hallmark of COVID-19, leading to severe illness, i.e. coagulopathy, multi-organ failure. FX06, a fibrin-derived peptide naturally occurring in the human body, formerly known as Bβ_15-42_, is a promising therapeutic candidate for endothelial complications like capillary leakage in COVID-19 and other forms of acute respiratory disorders. The aim of this project is to investigate whether FX06 can attenuate COVID-19 cytokine-triggered inflammatory processes *in vitro*.

**Methods:**

To mimic the inflammatory status of COVID-19, a human pulmonary microvascular endothelial cell line (ECs) – HULEC-5a, was treated with a cytokine cocktail comprised of ten different cytokines or chemokines at concentrations found in serum profiles of COVID-19 patients with severe illness, further referred to as the severe cytokine cocktail. ECs were treated with the severe cytokine cocktail for 24 h, in the absence or presence of FX06 for 2 h.

**Results:**

The severe cytokine cocktail enhanced peripheral blood mononuclear cell (PBMC)-endothelial adhesion and monolayer transmigration. This deleterious effect was significantly reduced by FX06. FX06 was also shown to mitigate the cytotoxic activity of allogeneic CD8^+^ T cells, which increased upon cytokine treatment. FX06 restored continuous vascular endothelial (VE)-cadherin/CD144 distribution on the EC surface and reversed morphological changes mediated by the severe cytokine cocktail, such as the elongation of F-actin stress fibers. FX06 reduced capillary-like structure formation of the severe cytokine cocktail treated-ECs, indicating FX06 down-regulated the pro-inflammatory angiogenic activity caused by the severe cytokine cocktail. Additionally, FX06 might assist in maintaining the normal barrier function of ECs by altering the surface expression of Syndecan-1 (SDC1/CD138). Proteomics and phosphoproteomics analyses demonstrated that FX06 in the presence of the severe cytokine cocktail inactivated RhoGTPase, which was confirmed by western blotting that FX06 attenuated RhoA, a member of RhoGTPase, enhanced by the severe cytokine cocktail and down-regulated the expression of the phosphorylated downstream protein, ROCK1.

**Conclusion:**

Overall, FX06 shows promising potential in normalizing ECs and reducing vascular leakage to protect the endothelium against the proinflammatory effect of COVID-19-triggered cytokines.

## 1 Introduction

COVID-19 disease has caused more than 7.1 million death cases, and hundreds of deaths every week have been reported to WHO until now. Although the World Health Organization (WHO) announced the end of the COVID-19 pandemic as a global health emergency, some COVID-19 patients after infection still suffer from long COVID, experiencing low exercise capacity and systemic metabolic disturbances (1). Blood–brain barrier (BBB) function impairment has been observed in long COVID patients, which is associated with cognitive impairment (brain fog) (2).

Cimino et al. reported that COVID-19 patients likely develop endothelial dysfunction (3). ECs abundantly express angiotensin-converting enzyme 2 (ACE2) receptor and SARS-CoV-2 directly infects the endothelium via the interaction between SARS-CoV-2 spike protein S1 (S1) and ACE2 receptor, which hampers ECs normal functions, such as anti-thrombosis, anti-inflammatory and microcirculatory homeostasis (4). In addition, SARS-CoV-2 infection leads to enhanced levels of cytokines and chemokines which have been found in sera of COVID-19 patients (5–9). Such a hyperinflammatory condition causes endothelial cell apoptosis, increased permeability, and loss of antioxidant defense (10–12). Hence, COVID-19 is regarded as also being a (micro)vascular and endothelial disease (13). Endothelial dysfunction contributes to these severe complications, such as coagulopathy, respiratory failure, acute respiratory distress syndrome (ARDS), multiorgan failure, and death (10, 14).

Therefore, protecting endothelial cells could be a promising therapeutic option, to avoid, or at least attenuate severe complications in COVID-19 patients. FX06, a fibrin-derived natural peptide, formerly known as Bβ_15-42_, is a potential therapeutic candidate for endothelial complications in COVID-19. FX06 has shown protective effects in both *in vitro* and *in vivo* studies. Wu et al. studied idiopathic systemic capillary leak syndrome (ISCLS) and found that FX06 inhibits the permeabilizing effects of sera from ISCLS patients *in vitro* (15). Gröger et al. studied mouse models with Dengue shock syndrome (DSS) that featured a progressive loss of endothelial barrier function, and the capillary leakage decreased and the survival rate significantly increased after FX06 treatment (16). Bergt et al. found that FX06 exhibited protective effects on reducing ischemia/reperfusion injury in pig and mouse models (17). Wolf et al. reported one Ebola-infected patient with improvement of vascular leak syndrome and a decrease in plasma viral load during supportive therapy with FX06 (18). FX06 increases oxygenation ability of patients with severe COVID-19-associated ARDS (19), and is currently in a phase 2 clinical trial for COVID-19 (20).

However, the protective MOA of FX06 against endothelial dysfunction is still not fully understood. Studies have shown that FX06 prevents disruption of the inter-endothelial junction protein, VE-cadherin, however, whether FX06 changes the abundance of VE-cadherin itself is not clear (15, 21, 22). One hypothesis suggests that FX06 competes with the E1 fragment of fibrin for interaction with VE-cadherin (23, 24). This E1 fragment is involved in promoting leukocyte transmigration through the endothelium by interacting with CD11c integrin on the leukocyte membrane and VE-cadherin, which forms adhesion junctions required for maintenance of the endothelial barrier (23, 25). However, FX06 cannot compete with VE-cadherin, due to its very low affinity to VE-cadherin compared to the E1 fragment. The E1 fragment potentially exists in the blood as a part of a complex, which prevents it from interacting with CD11c (26).

FX06 prevents cells from thrombin-induced cell activation, with the assistance of Fyn, a Src kinase family member, which has been found to play a role in VE-cadherin-mediated signaling (16). FX06 dissociates Fyn from VE-cadherin and associates Fyn with p190RhoGAP, the antagonist of RhoA. The activated p190RhoGAP promotes the formation of its GDP-bound form (27). The inhibition of RhoA activation contributes to the maintenance of barrier function and the prevention of cell contraction. The interaction between fibrin and the very-low-density lipoprotein (VLDL) receptor, prevents the dissociation of Fyn from VE-cadherin. FX06, in turn, inhibits VLDLR signaling, allows the dissociation of Fyn, inhibiting RhoA, thus reducing the transmigration of leukocytes through the endothelial barrier (26).

We aimed to investigate whether FX06 can be a potential candidate for COVID-19 patients experiencing endothelial dysfunction caused by COVID-19-triggered cytokines, and to better understand the MOA of FX06. ECs were treated *in vitro* with a cocktail of cytokines mimicking the inflammatory status of COVID-19 *in vivo* in the absence or presence of FX06. We found that FX06 restores the integrity of the vascular lining by reducing transendothelial migration (TEM) of immunological effector cells through human lung microvascular endothelial cells. FX06-treated ECs display continuous VE-cadherin distribution. Further, FX06 affects CD138, a component of glycocalyx, which could assist in reducing inflammation. Similarly, FX06 has been shown to decrease the angiogenic activity, thus reducing inflammation. FX06 increases activity of tight and gap junctions, decreases the protein abundance of TNFRSF17 and CD47, and reduces the phosphorylation of proteins related to RhoGTPases. FX06 down-regulates CSNK1A1 at all treatment time points making it an interesting target for future studies. Overall, these findings align with existing knowledge about FX06 action and endothelial barrier protection while providing new insights into its MOA that can potentially be exploited in the clinical treatment of endothelial dysfunction in numerous severe pathological conditions other than COVID-19.

## 2 Materials and methods

### 2.1 Cell culture

Human lung microvascular endothelial cell (HULEC-5a), purchased from American Type Culture Collection (ATCC, UK) were cultured in MCDB 131 media (cat#10372-019, Thermo Scientific, USA), supplemented with 15% Fetal Bovine Serum (FBS) (cat#A4766801, Thermo Scientific, USA), 1 µg/mL hydrocortisone (cat#H0396, Sigma-Aldrich, USA), 4% L-Glutamine (cat#25030-024, Thermo Scientific, USA), and 10 ng/mL Epidermal Growth Factor (EGF) (cat#10605-H01H, Sino Biological, China).

HULEC-5a were sent to Eurofins for a Cell Line Authentication Test and scored 100% of authenticity (as shown in **Supplementary Figure S1**).

### 2.2 The treatment design

To mimic the inflammatory status of SARS-CoV-2 infection in ECs, HULEC-5a were treated with a cytokine cocktail. This cocktail consists of a combination of 10 different cytokines or chemokines that were reported widely in COVID-19 patients’ serum profiles by other researchers (5–9). Concentrations of each cytokine or chemokine in the severe cytokine cocktail were designed based on their concentrations in serum samples of COVID-19 patients with severe illness (shown in **Table 1**). Fifty µg/mL FX06 (F4 Pharma GmbH, Austria) was used for experiments. HULEC-5a were treated for 24 h with fresh medium or the severe cytokine cocktail in the absence or presence of 50 µg/mL FX06 for 2 h as detailed in **Supplementary Figure S2**.

**Table 1:** Recipes of the severe cytokine cocktail, used to mimic the inflammatory status of COVID-19 patients with severe illness.

Cytokine name	Company	Cat no.	Working concentration, ng/mL
IL-1 Alpha	Sino Biological	10128-HNCH	20
IL-1 Beta	Sino Biological	10139-HNAE	10
IL-6	Sino Biological	10395-HNAE	50
IL-8	Sino Biological	10098-HNAE	20
TNF-Alpha	Sino Biological	10602-HNAE	100
IL-10	Sino Biological	10947-HNAE	20
IL-18	Sino Biological	10119-HNCE	50
IL-1 RA	Sino Biological	10123-HNAE	50
IP-10	Sino Biological	10768-HNAE	150
IFN-gamma	Sino Biological	11725-HNAS	50

### 2.3 Collagen-based TEM under static conditions

The collagen-based TEM assay under static conditions was performed in 96-well µ-plate black plates (cat#89626, IBIDI, Germany). On day 1, wells of the 96-well plate was first coated with a solution of 5 mg/mL Collagen I (cat#3440-100-01, R&D Systems, USA) in phosphate-buffered saline (PBS), and incubated for 2 h at 37°C. One-hundred µL of Collagen gel solution (Cultrex Rat Collagen I, NaHCO_3_, PBS, H_2_O, HULEC-5a media) was added into each well. The plate was centrifuged at 300 revolutions per minute of rotor (RPM) for 10 minutes to flatten the surface, then incubated for 1.5-2 h to polymerize.

ECs were labeled with CellTracker Orange (cat#C2927, Invitrogen, USA) for 20 minutes. Sixty thousand cells were seeded on the top of the collagen gel of each well.

Two days after seeding ECs, ECs formed a monolayer and a tight cell-to-cell contact. Fresh medium containing cytokine cocktails were added to ECs monolayer in the absence or presence of FX06, and incubated for 24 h.

After the 24 h-treatment, PBMCs were thawed in RPMI1640 medium (1% L-Glutamine, 1% Penicillin-Streptomycin (Pen-Strep), 10% FBS), and treated with 30 units/mL DNase 1 (cat#11284932001, Merck, Germany) for 1h, to avoid cell clumping. PBMCs were then labeled with CellTracker Green (cat#C7025, Invitrogen,USA) for 20 minutes. One million PBMCs were added to each well, and incubated for 18 h. The plate was imaged on a Phenix Opera High Content Screening (HCS) Confocal Microscope (Revvity, USA), and data was analyzed on Harmony software (Revvity, USA).

### 2.4 Collagen-based TEM under shear stress

Collagen-based TEM under shear stress was conducted in µ-Slide I Luer 3D slides (cat#87176, IBIDI, Germany). Slides were first coated with 5 mg/mL Collagen I in PBS (cat#3440-100-01, R&D Systems, USA), and incubated for 2 h at 37°C. Sixteen µL of Collagen gel solution (Cultrex Rat Collagen I, NaHCO_3_, PBS, H_2_O, Medium) was added into each chamber and then incubated for 1 h to polymerize.

HULEC-5a were labeled with CellTracker Orange (cat#C2927, Invitrogen, USA) for 20 minutes. HULEC-5a were diluted into 1.6 million cells/ml, and 150 µL of cell suspension was added into each slide.

After HULEC-5a formed a monolayer and a tight cell-to-cell contact, they were treated with fresh, medium, and severe cytokines for 24 h in the absence or presence of 2 h-treatment of FX06.

After 24 h-treatment, PBMCs were thawed in RPMI1640 medium (1% L-Glutamine, 1% Pen-Strep, 10% FBS), and treated with 10 units/µL DNase 1 (cat#11284932001, Merck, Germany) for 1 h, to avoid cell clumps. PBMCs were then labeled with CellTracker Green (cat#C7025, Invitrogen, USA) for 20 minutes. Slides were connected to the pump system (Serial number#0591, IBIDI, Germany) as shown in **Supplementary Figure S3**. Reservoirs were filled with PBMCs at a concentration of 1 million cells/mL. The shear stress with a flow rate of 0.4 dyn/cm^2^ was applied for 18 h.

Slides were imaged on Olympus FV1000 (Evident, Tokyo, Japan) and data was analyzed via CellProfiler 4.2.4.

### 2.5 Western blot analysis

HULEC-5a were lysed with 125 µL of ice-cold immunoprecipitation (IP) buffer (20 mM Tris HCL, 150 mM NaCl, 1 mM MgCl_2_, 1% Triton-X100). A Pierce bicinchoninic acid (BCA) assay (cat#23225, Thermo Scientific, USA) was performed in accordance with the manufacturer’s guidelines to quantify protein concentrations. Five µg of proteins and 5 µL of prestained molecular weight ladder were added to corresponding wells in Bolt™ Bis-Tris Plus Mini Protein Gels (cat#NW04125BOX, Invitrogen, USA). Following transfer onto polyvinylidene difluoride (PVDF) membrane, the membrane was blocked for 1 hour with 5% milk in Tris Buffered Saline with Tween 20 (TBS-T) at room temperature. The membrane was then incubated with anti-RhoA Antibody, (cat#sc-418, Invitrogen, USA) (1:1000 in 5% BSA in TBST) overnight at 4°C. The membranes then underwent incubation with the secondary antibody, anti-mouse IgG, horseradish peroxidase (HRP)-linked Antibody, (cat#7076P2, Cell Signaling Technology, USA) (1:5000 in 5% milk in TBST) for 1 hour on a shaker at room temperature. SuperSignal™ West Femto Maximum Sensitivity Substrate (cat#34095, Thermo Scientific, USA) was applied, and the membranes were imaged using the iBright imaging system (Thermo Scientific, USA).

### 2.6 Immunofluorescence for 2D culture cells

For the measurement for surface marker expression, 4% paraformaldehyde (PFA) solution was applied to fix cells at room temperature for 15 minutes. Imaging for total expression requires both fixation and permeabilization. ECs were incubated with acetone with 12-minute incubation at -20°C. HULEC-5a were then washed 3 times with PBS (5 minutes each time).

Cells were incubated with 10% goat serum in PBS for 1 h, to avoid nonspecific binding. Regarding CD144 surface imaging, the primary antibody solution, mouse Anti-Human CD144 (cat#555661, BD Biosciences, USA), was incubated overnight at 4°C. After 3 washes with PBS, secondary antibody goat anti-mouse Alexa Fluor 546 (cat#A11003, Invitrogen, USA) was added and incubated for 1 h at 37°C. In terms of surface and total CD138 imaging, the primary antibody solution, CD138 Recombinant Antibody (JM11-21), rabbit monoclonal (cat#MA5-32600, Thermo Scientific, USA), was incubated overnight at 4°C. After 3 washes with PBS, secondary antibody goat anti-rabbit Alexa Fluor 488 (cat#A11008, Invitrogen, USA) was added and incubated for 1 h at 37°C. Regarding F-actin imaging, ECs were incubated with Alexa Fluor™ 488 Phalloidin (cat#A12379, Invitrogen, USA) for 1 h at room temperature. At the end, cells were counterstained with 600 nM DAPI (cat#32670-5MG-F, Sigma-Aldrich, USA) for 15 minutes at 37°C.

Imaging was conducted by Revvity Phenix Opera HCS Confocal Microscope using 40x water immersion objective, or performed using Olympus FV1000, with 40x objective. Images were analyzed using Revvity Harmony software or ImageJ.

### 2.7 Flow cytometry for the measurement of apoptosis and necrosis

ECs harvested from culture was washed with PBS at 300 relative centrifugal force (RCF/g) for 5 minutes at 4°C. ECs were then resuspended in Fluorescence-activated cell sorting (FACS) buffer (2 mM EDTA, 0.5% BSA in PBS) at the concentration of 1 million cells/mL.

Regarding the measurement for apoptosis and necrosis, 3 µL of Oxazole Yellow/YOPRO-1 at the concentration of 0.001 mM/mL (cat#Y3603, Invitrogen, USA) was added to 300 µL of cell suspension and incubated for 10 minutes at 4°C. Three µL of Propidium iodide (PI) at the concentration of 1 µg/mL (cat#P4864, Sigma-Aldrich, USA) was added into each sample before running on the CytoFLEX Flow Cytometer (Beckman, USA). The minimum information for this flow cytometry experiment following the Minimum Information about a Flow Cytometry Experiment (MIFlowCyt) standard was provided in **Supplementary Table S1** (28).

### 2.8 Enzyme-linked immunosorbent assay for shed CD138

The Human Syndecan 1 (SDC1) ELISA Kit (cat#EHSDC1, Invitrogen, USA) were applied to measure the shedd CD138. After the treatment, cell culture supernatant from each well was collected for ELISA as per manufacturer’s instructions. One-hundred µL of standards and undiluted samples were added to the Human Syndecan 1 Antibody Coated wells, and incubated overnight at 4°C with gentle agitation. After antigen binding, wells were subsequently incubated with biotin conjugate, Streptavidin-HRP, 3,3’,5,5’-Tetramethylbenzidine (TMB) substrate, and the absorbance measured at 450 nm on a SpectraMax ^®^ M microplate reader (Molecular Device). after adding the stop solution. The concentration values were calculated through the standard curve based on the reading values from technical duplicates for each sample.

### 2.9 T cell based cytotoxicity assay

ECs was inactivated mitotically by Mitomycin C (MMC, cat#73274, Stemcell Technologies, Canada) at a concentration of 20 µg/mL for 2 h. CD8^+^ T cells were isolated from PBMC through the magnetic-activated cell sorting (MACS) CD8^+^ T Cell Isolation Kit (cat#130-096-495, Miltenyi Biotech, Germany). Followed by isolation, CD8^+^ T cells were resuspended in IL-2 medium, which is RPMI1640 medium containing 1% L-Glutamine, 1% Pen-Strep, 10% heat-inactivated human serum (cat#H3667, Sigma-Aldrich, USA) and 300 U/mL IL-2 (cat#200-02, Thermo Scientific, USA). Mitomycin C-inactivated HULEC-5a were collected and seeded together with CD8^+^ T cells at the ratio 1:1 for 7 days.

Two days before performing the cytotoxicity assay, the targets (HULEC-5a) were collected and labeled with 10 µL of 3 mM 3,3’-Dioctadecyloxacarbocyanine perchlorate (DiO) (cat#D275, Thermo Scientific, USA) at 1 million cells per ml. Cells were seeded into T25 flasks. ECs were then treated with medium, the severe cytokine cocktail with or without FX06, respectively. The next day the cell suspension from different treatment groups was harvested, and mixed with stimulated CD8^+^ T cells (effectors). Targets and effectors were mixed at the ratio of 20:1, 10:1 and 5:1, and incubated for 4 h.

Followed by the incubation, all cell suspension was collected. CytoFLEX Flow Cytometer was utilized to measure the cell lysis caused by CD8^+^ T cells. The experimental description on flow cytometry was provided in **Supplementary Table S2**, based on the MIFlowCyt standard (28).

### 2.10 Statistical analysis

Image visualization and data quantification were performed via Revvity Harmony software, Cellprofiler 4.2.4 or ImageJ. The quantitative assessments for Western Blotting were conducted by ImageJ software. Gatings for flow cytometry analysis were done on CytExpert 2.5.

For comparisons between two groups, Student’s t-tests were applied. In cases where multiple groups were compared, statistical analysis for results of experiments were conducted using one-way ANOVA followed by multiple comparison tests with Tukey on Graphpad Prism 9.0. Statistical significance was confirmed with a p-value < 0.05. Bar charts were plotted in Graphpad Prism 9.0.

### 2.11 Mass spectrometry analysis

#### 2.11.1 Proteomic sample preparation and mass spectrometry

To prepare the samples for proteomic analysis, 100 µL of an ice-cold lysis buffer containing 8 M Urea and 50 mM Tris HCL was added to resuspend each cell pellet. The samples were then sonicated 2 x 9 seconds at 15% power using Ultrasonic Homogenizer (Syclon, Ningbo, China), followed by a BCA assay (cat#23225, Thermo Scientific™, Waltham, Massachusetts, United States) to ensure proper protein concentration. 100 µg of proteins were employed for mass spectrometry sample preparation. The protein samples were subjected to reduction by the addition of 8 mM dithiothreitol (DTT) and carboxylation by adding 20 mM iodoacetamide. These steps were carried out on an Eppendorf Thermomixer (1000 rpm at 30°C for 30 min) for protein modification while avoiding light exposure due to iodoacetamide’s photosensitivity. The samples’ urea concentration was subsequently reduced to below 2 M by dilution with 50 mM Tris HCL. Protein digestion was achieved by introducing sequencing-grade trypsin at a 1:50 enzyme-to-protein ratio, and the digestion process occurred overnight with gentle shaking on a Thermomixer (850 rpm, 37°C). The digestion process was subsequently terminated by adding formic acid to reach a final concentration of 1%.

The samples were then cleaned using C18 HyperSep SpinTips (cat#87784, Thermo Scientific™, Waltham, Massachusetts, United States). The SpinTips were conditioned with 200 µL of elution buffer (60% acetonitrile/ACN in 0.1% trifluoroacetic acid/TFA) and equilibrated with 200 µL of equilibration and washing buffer (0.1% TFA in LC-MS grade water). 100 µg of digested samples were loaded onto the SpinTips, and the process involved several washing and elution steps as per the manufacturer’s instructions. Following sample cleanup, the samples were analysed using a Bruker Tims TOF Pro mass spectrometer (Billerica, Massachusetts, United States) connected to a Evosep One liquid chromatography system.

#### 2.11.2 Data preprocessing

The mass spectrometer raw files were analysed using the DIA-NN software (29). The row files were searched against the Homo sapiens Swissprot reference proteomes database (Version 1.8.1). LFQ intensities were log2-transformed. Proteins/phosphosites with missing values in more than 50% of samples per group were dropped. Missing values were imputed using the group mean imputation with normal distribution correction and tail-based imputation methods using the PhosR package (30). The phosphoproteomics data was normalised using the proteomics data by fitting the linear model.

#### 2.11.3 Differential expression analysis

Differential expression analysis for proteins/phosphosites was conducted using limma by fitting linear models for each protein/phosphosite using the least squares method (31). Only the proteins/phosphosites with adjusted p-values less than 0.05 and fold changes greater than 1.5 or less than -1.5 were considered as statistically significant changes. The over-representation analysis (ORA) was performed using g:Profiler to annotate processes in which upregulated and downregulated proteins could be involved in (32).

#### 2.11.4 Pathway analysis

Pathway analysis was performed using Gene Set Enrichment Analysis (GSEA) based on lists of pathways from the Kyoto Encyclopedia of Genes and Genomes (KEGG) database and a vector of gene-level t-statistics retrieved from the differential expression analysis using msigdbr and fgsea libraries (33, 34). As a result of GSEA, a list of enriched pathways with their normalised enrichment scores and adjusted p-values was obtained. Only the pathways with adjusted p-values less than 0.05 were selected for further review.

#### 2.11.5 Kinase enrichment analysis (proteomics data)

Kinase Enrichment Analysis (KEA) was performed using the KEA3 library (35). Differentially expressed proteins were used as input for this analysis. The top 10 kinases by enrichment scores, retrieved from the KEA analysis for each cell group comparison, were selected for further review.

#### 2.11.6 Kinase substrate enrichment analysis (phosphoproteomics)

Kinase Substrate Enrichment Analysis (KSEA) was performed by scoring kinases based on changes in phosphorylation of differentially phosphorylated sites and the Kinase Substrate Dataset from PhosphoSitePlus (https://www.phosphosite.org/staticDownloads) using the KSEA App R package (36). P-values and log2 fold change values from the phosphosites differential expression analysis were used as input. Only kinases with p-values less than 0.05 and a number of kinase substrates more than 2 were selected for further review.

#### 2.11.7 Drug similarity analysis

Drug Similarity Analysis was performed by inputting statistically significant differentially expressed proteins into L1000CDS² search engine (https://maayanlab.cloud/L1000CDS2/#/index) based on the LINCS database (37). Top 15 drugs by similarity score for each cell group comparison were selected for further review.

#### 2.11.8 Mfuzz clustering

Soft clustering was performed on proteomics and phosphoproteomics data to identify clusters, which could be influenced by the FX06 drug, using the Mfuzz package (38). Intensities of technical replicates were averaged. Mfuzz clustering was performed with parameters c = 32 and m = mestimate(mfuzz input). Only the clusters with noticeable changes in protein and phosphoprotein abundance, which are caused by the effect of cytokine cocktail and reversed by the drug to the levels of control samples, were selected for further review. The over-representation analysis performed using g:Profiler was used to annotate processes, in which proteins of each cluster could be involved in (32).

#### 2.11.9 DIABLO analysis

DIABLO (Data Integration Analysis for Biomarker discovery using Latent Components) was performed with the mixOmics R package to integrate proteomics and phosphoproteomics data (39). This analysis was performed with 5 components, allowing the model to capture key latent variables across the datasets. The number of features was selected based on the model performance, tested with 5-fold cross validation. To identify biological processes associated with the most influential features, over-representation analysis was performed using g:Profiler. This analysis focused on the top 100 proteomics features and top 100 phosphoproteomics features, selected based on their absolute component weights in the DIABLO model. The resulting annotations provide insight into the key biological processes potentially affected by these high-impact features.

## 3 Results

### 3.1 FX06 protects human pulmonary microvascular ECs against the severe COVID-19 cytokine-triggered TEM of PBMC under both static and shear stress conditions

**Figure 1:**
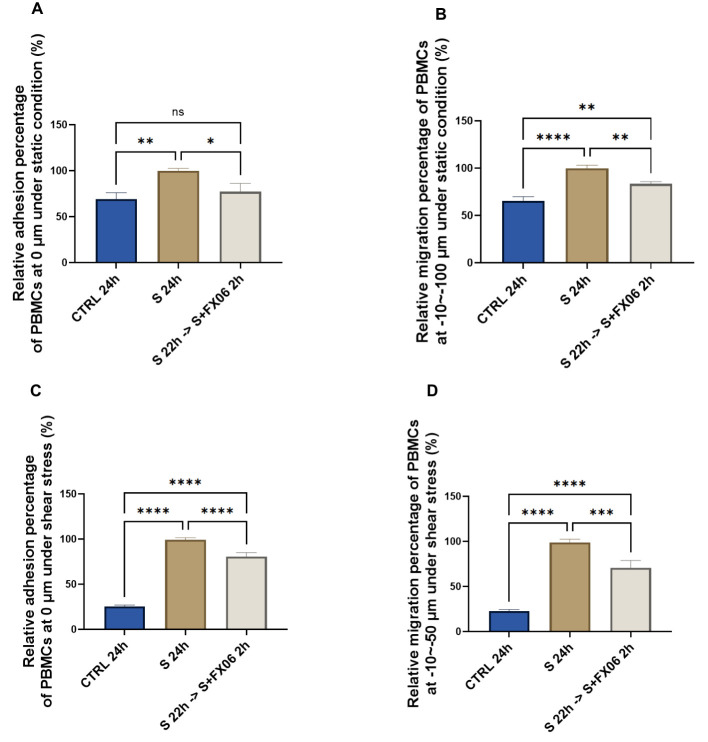
The effects of the severe cytokine cocktail and FX06 on TEM through EC monolayer. PBMC adhesion (0 μm) under static conditions **(A)** and under shear stress conditions **(C)**, PBMCs migration (-10--100 μm) under static conditions **(B)** and (-10--50 μm) under shear stress conditions **(D)** from three independent experiments were measured with the Revvity Harmony software. FX06 reduced the increased TEM caused by the severe cytokine cocktail in ECS cultured under both static and shear stress conditions. One-way ANOVA, ns: non-significant, *P<0.05, **P<0.01, ***P<0.001, ****P<0.0001.

FX06 has shown to reduce vascular leakage and maintain the integrity of ECs (15, 16), therefore we investigated whether FX06 has the same ability to protect ECs against COVID-19-triggered cytokines. For that matter, ECs were treated for 24 h with medium or the severe cytokine cocktail in the absence or presence of FX06 for the final 2 h. FX06 alone does not affect the viability of ECs (**Supplementary Figure S4**). A 24 h incubation with the severe COVID-19-triggered cytokine cocktail significantly increased the adhesion of PBMCs to the endothelium, which was reversed by the 2 h FX06 treatment (**Figures 1A, C**). The severe cytokine cocktail also enhanced the migration of PBMC through the endothelium, and 2 h-treatment of FX06 in the presence of cytokines partially restored the integrity of the vascular lining (**Figures 1B, D**).

### 3.2 FX06 exerts a protective effect against the enhanced TEM triggered by the addition of S1 protein to the severe cytokine cocktail

**Figure 2:**
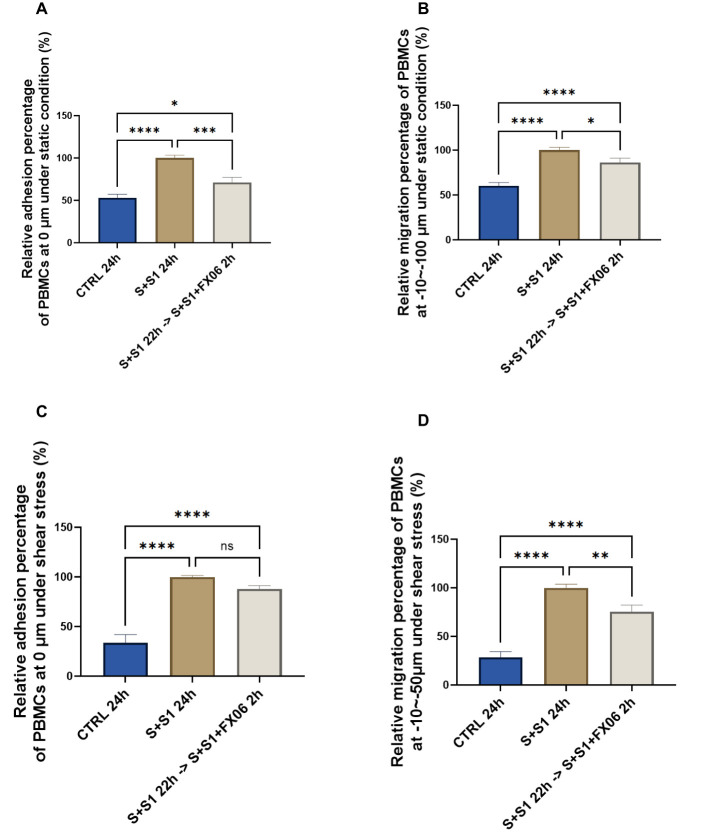
The protective effects of FX06 in ECs treated with the severe cytokine cocktail and S1. PBMC adhesion (0 μm) and migration (-10--100 μm) under static conditions **(A, B)**, PBMC adhesion (0 μm) and migration (-10--50 μm) under shear stress conditions **(C, D)**, from three independent experiments, were measured on Revvity Harmony software. The severe cytokine cocktail together with S1 significantly increased the TEM through ECs monolayer, and FX06 significantly reduced the TEM. One-way ANOVA, ns: non-significant, *P<0.05, **P<0.01, ***P<0.001, ****P<0.0001.

SARS-CoV-2 interacts with ACEs on ECs via S1 protein, therefore FX06 is further investigated whether it displays any protective effects on ECs under both COVID-19-trigered cytokines and the influence of S1 (40). ECs were treated for 24 h with medium, the severe cytokine cocktail plus S1 in the absence or presence of FX06. Addition of S1 to the cytokine cocktail significantly increased the adhesion and the migration of PBMCs to and through the ECs in static (**Figures 2A, C**) and shear stress conditions (**Figures 2B, D**) to a slightly greater extent than for the cytokine cocktail alone. Treatment with FX06 failed to prevent PBMCs adhesion to ECs when S1 protein was added to the severe cytokine cocktail under shear stress (**Figure 2C**). However, FX06 was still showing protective effect against PBMCs adhesion under static condition (**Figure 2A**) and against PBMCs migration through the endothelium both under static and shear stress conditions (**Figures 2B, D**). More TEM was triggered by the addition of the S1, FX06 still displays a significant protective effect on the endothelial barrier.

### 3.3 The protective effect of FX06 on ECs is not related to an anti-apoptotic or an anti-necrotic effect

**Figure 3:**
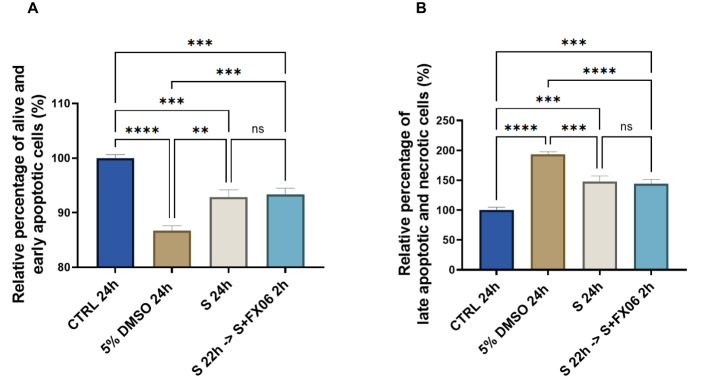
The effects of the severe cytokine cocktail in the absence or presence of FX06 on the viability of ECs. Statistical analysis was conducted on the percentage of alive and early apoptotic cells **(A)**, and the percentage of late apoptotic and necrotic cells **(B)** from three independent flow cytometric experiments. The severe cytokine cocktail significantly decreased the percentages of alive and early apoptotic cells and increased the percentages of late apoptotic and necrotic cells. FX06 did not reverse the changes caused by the severe cytokine cocktail. One-way ANOVA, ns: not significant, *P<0.05, **P<0.01, ***P<0.001, ****P<0.0001.

Whether FX06 prevents TEM of PBMCs through ECs by inhibiting cell death to facilitate the formation of more integrated endothelial barriers, becomes of interest. ECs were treated for 24 h with medium or the severe cytokine cocktail in the absence of FX06, with the presence of FX06 in the last 2 h. Five percent DMSO served as a positive control. The results showed that 24 h-treatment of the severe cytokine cocktail increased the percentage of late apoptotic and necrotic cells and decreased the percentage of alive and early apoptotic cells (as shown in **Figures 3A, B**).

Two-hour treatment of FX06 in the severe cytokine cocktail does not decrease apoptosis and necrosis in ECs. The results indicate that FX06 does not have anti-apoptotic nor anti-necrotic effects. FX06 does not protect the endothelium from apoptosis and necrosis caused by COVID-19-triggered cytokines, suggesting alternative mechanisms of action of FX06.

### 3.4 FX06 might assist in maintaining the normal barrier function of ECs by altering the surface expression of syndecan-1

**Figure 4:**
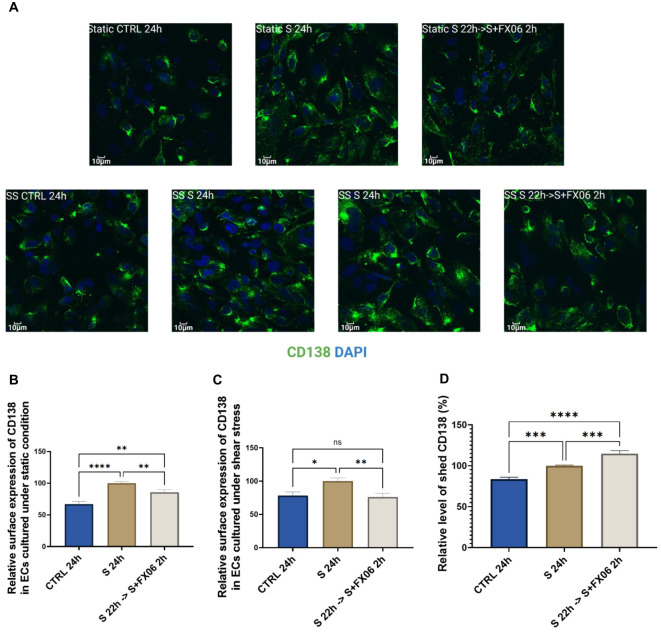
The surface expression of CD138 in ECs, treated for 24 h with medium, the severe cytokine cocktail in the absence or presence of 2 h-treatment of FX06. Images of CD138 **(A)** showed the surface marker in ECS cultured under static and shear stress conditions. Images were taken on Evident Olympus FV1000 with 40x objective. The surface expression of CD138 in ECs cultured under both static and shear stress conditions **(B, C)** was measured via immunofluorescence. Under both static and shear stress conditions, the severe cytokine cocktail caused more surface expression of CD138, and FX06 reversed the surface expression level of CD138 back to control levels. **(D)** shows the level of shed CD138. The severe cytokine cocktail induced more shed CD138, whereas FX06 treatment caused even more. One-way ANOVA, ns, not significant, *P<0.05, **P<0.01, ***P<0.001, ****P<0.0001.

Endothelial glycocalyx contributes to vascular barrier function, and a cytokine-triggered degradation of endothelial glycocalyx can further enhance inflammation (41). CD138 as a component of the endothelial glycocalyx has often been reported as a potential marker for the illness progression of COVID-19 patients (42).The expression of CD138 was measured in ECs treated for 24 h with medium, the severe cytokine cocktail in the absence or presence of 2h-treatment of FX06. The severe cytokine cocktail induced a higher surface expression of CD138 of ECs, which could facilitate more interaction between syndecan-1 and chemokines, thereby causing more PBMC migration. However, the surface expression of CD138 decreased after FX06 treatment and FX06 assisted in the resolution of inflammation (as shown in **Figures 4B, C**).

Soluble CD138 is considered to be a biomarker for the disease course in COVID-19 patients (43), thereby ELISA for soluble CD138 measurement was conducted. The severe cytokine cocktail caused an increased level of soluble CD138, compared to untreated ECs (**Figure 4D**). However, ECs treated with the severe cytokine cocktail in the presence of FX06 induced more soluble CD138 (**Figure 4D**), which might be the reason why FX06 treatment decreased the surface expression of CD138. But whether soluble CD138 can be a treatment prognostic marker for COVID-19 patients treated with FX06 remains to be investigated further.

Total expression of CD138 was measured and no significant changes were observed among groups (**Supplementary Figure S5**). The results indicate that ECs treated with the severe cytokine cocktail had higher surface expression and lower internal expression, while ECs treated with medium or the severe cytokine cocktail plus FX06 had lower surface expression and higher internal expression. Even though FX06 caused more shed CD138, ECs produced more CD138 to maintain the normal surface expression of CD138 as comparable to untreated ECs. Our results suggest that FX06 assists in normalising the endothelium regarding the glycocalyx of ECs. The severe cytokine cocktail induces a relocalisation of CD138 from the internal space to the plasma membrane. This shift in CD138 localisation is prevented by the addition of FX06 to the severe cytokine cocktail. The additional CD138 produced in the presence of the severe cytokine cocktail seems to be excreted in presence of FX06 to maintain surface expression close to untreated ECs.

### 3.5 FX06 reverses morphology changes caused by the severe cytokine cocktail and shortens the F-actin fiber length

**Figure 5:**
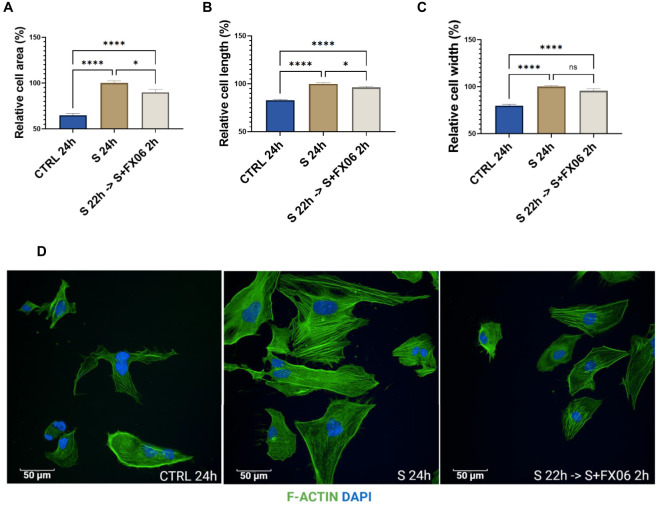
The morphological changes of ECs. The cell area **(A)**, cell length **(B)**, and cell width **(C)** were measured with the Revvity Harmony software. One-way ANOVA, ns: not significant, *P<0.05, ****P<0.0001. **(D)** ECs were treated with medium, the severe cytokine cocktail, and the severe cytokine cocktail in the presence of 2h-treatment of FX06. ECs were incubated with anti-F-actin antibodies, Alexa Fluor 488 Phalloidin (green). DAPI (blue) was utilised to stain nuclei. Images were taken with a Revvity Phenix Opera Confocal Microscope using 40x water immersion objective. Scale bar: 50 μm.

Morphological changes were observed in ECs treated with the severe cytokine cocktail. The severe cytokine cocktail significantly increased the cell area, length, and width of ECs (**Figure 5**). FX06 treatment decreased the cell size and reduced cell elongation. Only a slight decrease without significant differences in cell width was observed. It indicates that FX06 treatment significantly decreases the cell size mostly due to a reduction in the cell lengths with only a limited decrease of cell width.

F-actin fibers in ECs treated with the severe cytokine cocktail were more elongated compared to untreated cells, as an indication of stress. 2 h-treatment of FX06 in the presence of cytokines shortened F-Actin stress fibers.

### 3.6 FX06 limits the enhanced angiogenic activity induced by the severe cytokine cocktail

**Figure 6:**
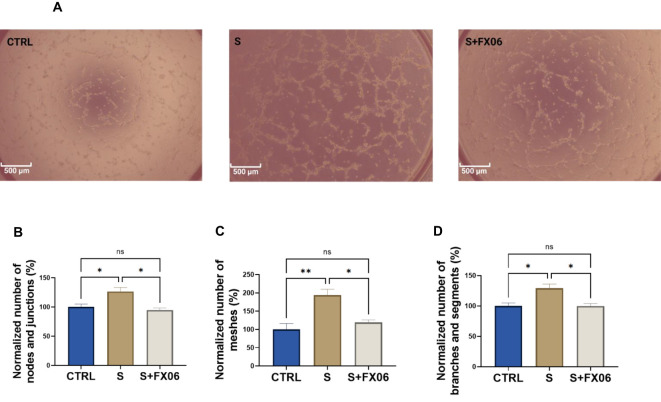
The effects of the severe cytokine cocktail and the severe cytokine cocktail with the presence of FX06 on angiogenesis activities in ECs. Images from **(A)** were taken from Zeiss Primovert microscope with a 4x objective. Scale bar: 500 μm. The total number of nodes and junctions **(B)**, the number of meshes **(C)**, and the total number of segments and branches **(D)**, from five independent experiments were measured by angiogenesis analyzer on ImageJ. One-way ANOVA, ns: not significant, *P<0.05, **P<0.01.

Proinflammatory cytokines, for instance, IL-1β, IL-6, and TNF-α, can promote angiogenesis (44). The angiogenic activity of ECs seeded on Matrigel™ and treated with medium, the severe cytokine cocktail, and the severe cytokine cocktail with FX06, was measured. The severe cytokine cocktail significantly promoted angiogenesis with the increased number of nodes and junctions, meshes, segments, and branches (**Figures 6B–D**). FX06 co-incubated with the severe cytokine cocktail decreased angiogenic activity. Cell morphology changes are associated with angiogenesis, and cell elongation is the first step of angiogenesis (45). Increased angiogenesis induced by the severe cytokine cocktail aligned with elongated cell length (**Figure 5B**).

### 3.7 FX06 restores continuous VE-cadherin/CD144 distribution on EC after disruption by the severe cytokine cocktail

**Figure 7:**
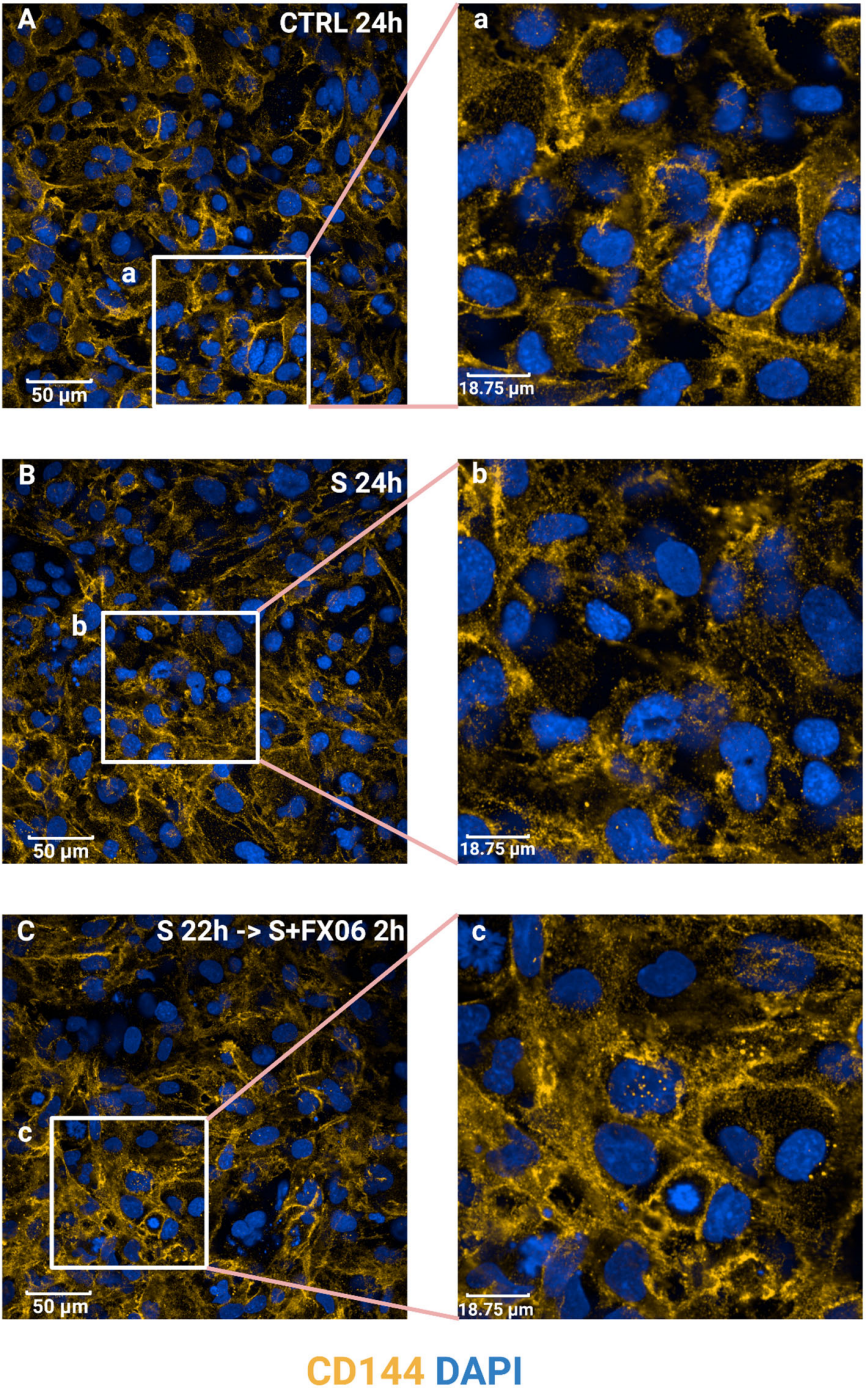
The effects of the severe cytokine cocktail and the severe cytokine cocktail with the presence of FX06 on VE-cadherin distribution in ECs. ECs were incubated with anti-VE-cadherin antibodies and stained with Alexa Fluor 546 (yellow). DAPI (blue) was utilised to stain nuclei. ECs were treated with medium, the severe cytokine cocktail, and the severe cytokine cocktail in the presence of 2 h-treatment of FX06. Images **(A-C)** were taken with a Revvity Phenix Opera Confocal Microscope using 40x water immersion objective. Scale bar: 50 μm. White boxes highlight the zoomed areas (a-c). Zoomed scale bar: 18.75 μm.

VE-cadherin junctions are connected between ECs and involved in maintaining EC integrity (46, 47). To study VE-cadherin junctions on ECs, ECs were treated for 24 h with medium, the severe cytokine cocktail in the absence or presence of 2 h-treatment of FX06. In ECs cultured under static conditions, the severe cytokine cocktail caused more discontinuous VE-cadherin junctions as shown in **Figure 7**. FX06 treatment facilitated ECs to form more continuous junctions.

### 3.8 The severe cytokine cocktail increases the allogenicity of ECs towards cell lysis by allogeneic CD8^+^ T cells, protective effect of FX06

**Figure 8:**
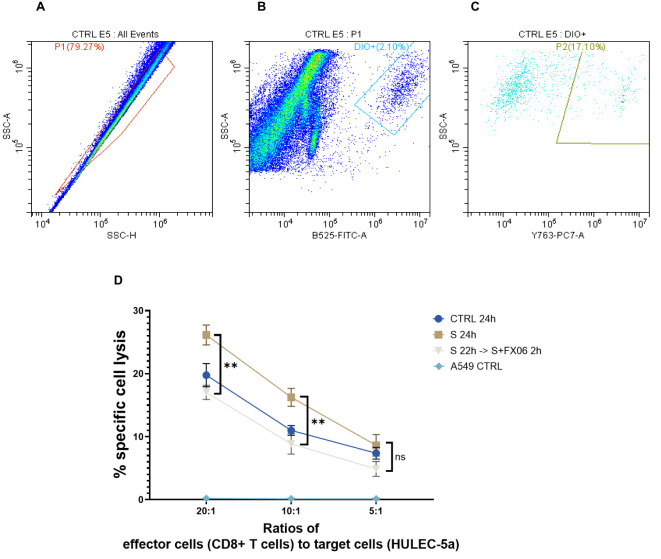
The cytotoxicity of CD8^+^ T cells on ECs. The percentages of ECs specific cell lysis caused by CD8^+^ T cells were measured by flow cytometry analysis. **(A-C)** The flow cytometry analysis gating strategy. The graphs were from one sample. First singlets were gated **(A)**, then DIO+ cells were gated in singlets **(B)**. At the end PI^+^ population that represented CD8^+^ T cells-induced EC lysis was gated in DIO+ cells **(C)**. Same gatings were applied to all samples with different treatment. **(D)** A549 CTRL served as a negative control. Severe cytokine cocktail treatment increased the cell lysis which was downregulated by FX06 treatment. One-way ANOVA, ns: not significant, **P<0.01.

To investigate whether the severe cytokine cocktail influences the immunogenicity of ECs, CD8^+^ T cells had been stimulated with ECs for 7 days. Subsequently, ECs as target cells were treated for 24 h with medium, the severe cytokine cocktail with or without FX06. The gating strategy was shown in **Figures 8A–C**. P1 in **Figure 8A** represented a single cell population. DiO+ population was target cells (ECs) in **Figure 8B**. P2 population represented EC lysis induced by CD8+ T cells in **Figure 8C**. The severe cytokine cocktail increased antigenicity of ECs as shown by a higher specific lysis in **Figure 8D**. FX06 treatment reduced the cytotoxic effects of CD8^+^ T cells.

### 3.9 Proteomics and phosphoproteomics data analysis

#### 3.9.1 Experimental design and significant changes in protein expression and phosphorylation patterns

**Figure 9:**
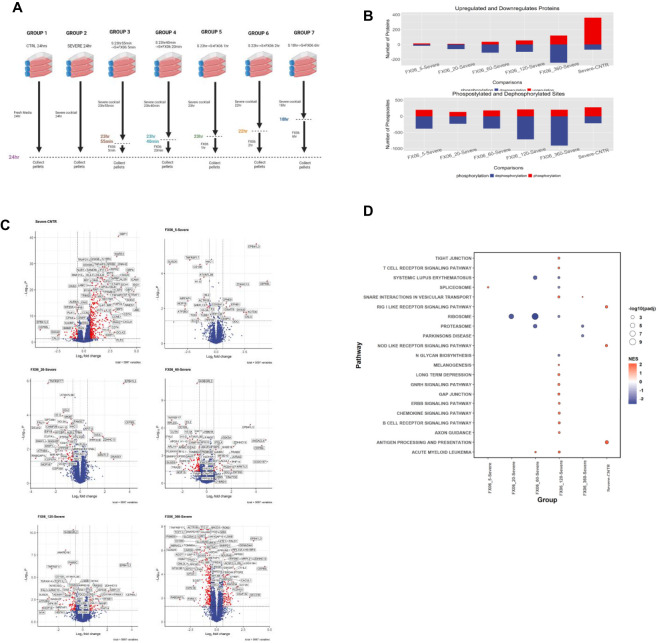
**(A)** The experimental design of proteomics and phosphoproteomics analysis. The illustration was created by Biorender. **(B)** Box plots with the overall number of upregulated and downregulated proteins (top) and phosphosites (bottom) for six cell group comparisons including FX06 treatments (5, 20, 60, 120, 360 min) versus Severe and Severe versus Control. **(C)** Volcano plots of differentially expressed proteins and red circles depict significantly differentially expressed proteins. FX06 induces differentially expressed proteins over the time. **(D)** Bubble plot with the results of Gene Set Enrichment Analysis using the KEGG database. The severe cytokine cocktail with the addition of 2h-FX06 shows the protective effects on EC barrier integrity.

LC-MS-based proteomics and phosphoproteomics analyses were performed to assess alteration in protein abundance and protein phosphorylation changes in a time series study, to investigate the MOA of FX06 at multiple treatment time points. Seven different treatment conditions were tested (**Figure 9A**) including control (CNTR), severe, and five FX06-treated groups with varying exposure times (5, 20, 60, 120, and 360 minutes). Control cells were incubated in fresh media for 24 hours and the Severe group was exposed to the severe cytokine cocktail for the same duration. The FX06-treated groups were first incubated with the severe cytokine cocktail and then treated with FX06 for their respective durations, maintaining a total incubation time of 24 hours.

Treatment durations were as follows:

- FX06_5: 23 hours 55 minutes with the severe cytokine cocktail, 5 minutes with FX06;
- FX06_20: 23 hours 40 minutes with the severe cytokine cocktail, 20 minutes with FX06;
- FX06_60: 23 hours with the severe cytokine cocktail, 60 minutes with FX06;
- FX06_120: 22 hours with the severe cytokine cocktail, 120 minutes with FX06;
- FX06_360: 18 hours with the severe cytokine cocktail, 360 minutes with FX06.

After incubation, cells from all groups were collected for mass spectrometry analysis.

The proteomics and phosphoproteomics analysis revealed significant changes in protein expression and phosphorylation patterns following FX06 treatment of endothelial cells exposed to the severe cytokine cocktail (**Figure 9B**). The number of differentially changed proteins and phosphosites increases over time in FX06-treated groups compared to cytokine-treated cells, indicating a progressive cellular response to FX06. The number of dephosphorylated sites increased in FX06-treated ECs, compared with the severe cytokine cocktail-treated ECs. But no major changes were observed regarding the number of phosphorylated sites.

#### 3.9.2 Proteomics analysis shows that FX06 increases the activity of tight and gap junctions, and reduces immune cell infiltration

The severe cytokine cocktail induces the upregulation of several proteins associated with the promotion of inflammatory responses and increased immune cell infiltration, including GBP1, WARS1, NFKB2, ICAM1, IFIT2, HLA-B, RELB, CXCL9, OAS2, and IL15RA (**Figure 9C**). The gene set enrichment analysis (**Figure 9D**) identifies that the severe cytokine cocktail induces the activation of the RIG I-like receptor and Nod-like receptor signaling pathways, as well as the antigen processing and presentation signaling pathway. FX06 counteracts cytokine-induced effects by reducing TNFRSF17 levels across all treatment periods and decreasing CD47 expression starting from the 20 minute time point. At the 2-hour time point, FX06 increases the activity of Tight and Gap Junction signaling, Gonadotropin-releasing hormone and ERBB signaling, which contributes to the EC integrity.

FX06 reverses protein abundance alterations caused by the severe cytokine cocktail, as shown in **Supplementary Figure S6**. This effect varies over time, with significant alteration of proteins related to cellular stress responses, cytoskeletal dynamics and other proteins that might collectively contribute to endothelial cell function and barrier integrity.

#### 3.9.3 Phosphoproteomics analysis reveals that FX06 triggered inactivation of RhoGTPase signaling

**Figure 10:**
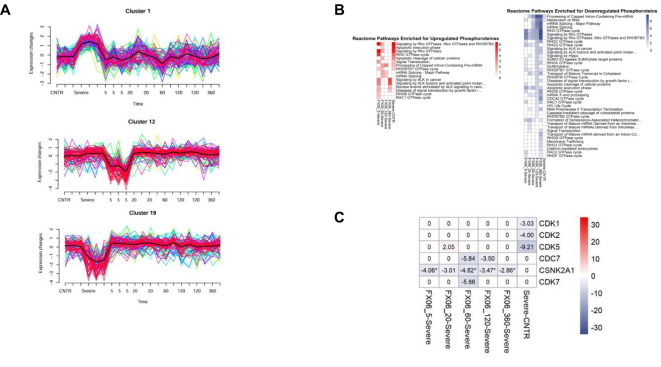
**(A)** MFuzz clustering. Line charts with changes in protein phosphorylation for each phosphorylation site in its respective cluster at each time point of the experiment. Lines with yellow, green, and blue colours indicate proteins with low membership values, while the lines with red and purple colours correspond to proteins with high membership values. The black line highlights the centre of the cluster at each time point of the experiment. The addition of FX06 to the severe cytokine cocktail has shown to normalize the changesinduced by the severe cytokine cocktail. **(B)** Heatmaps with the results of Over-representation analysis using the Reactome databases. The reversed effects of FX06 in Rho GTPase are observed. **(C)** Heatmap with Kinase Enrichment Scores from KSEA based on whole phosphoproteomics data. Kinases with Enrichment Scores with FDR values less than 0.05 are marked with the "*" symbol. Only kinases with p-value < 0.05 are selected. CSNK2A1 is downregulated by FX06 at all time points.

The reversion effect for FX06 also has been shown in the phosphoproteomics study. Mfuzz clustering analysis showed that FX06 can reverse changes on cytoskeletal changes caused by the severe cytokine cocktail. **Figure 10A** displays clusters that showed a significant change in phosphorylation levels induced by the severe cytokine cocktail and reversed FX06. Proteins from Clusters 1 and 19 that are reversed by FX06, are associated with focal adhesions. Additionally, proteins from cluster 19 are related to cadherin binding and cytoskeletal organization. Cluster 12 is associated with RhoGTPase signaling and it displays a dephosphorylation of RhoGTPase at 5-minute of FX06. A pathway analysis using phosphoproteomics data, also revealed inactivations of RhoGTPase in presence of FX06 (**Figure 10B**). To identify the upstream kinases responsible for changes in phosphorylation level, a kinase-substrate enrichment analysis was performed. The treatment of FX06 induces the inactivation of CSNK2A1 at all time points, starting from 5 minutes (**Figure 10C**), which can potentially facilitate the stabilization of the endothelial barrier via the Hedgehog signaling pathway (48). Based on the results from drug similarity analysis (**Supplementary Table S3**), the treatment of FX06 is found to be similar to multiple inhibitors. BMS-536924, an Insulin-like growth factor 1 receptor inhibitor, showed similarity to FX06 at 5 minutes, 20 minutes, 60 minutes, and 2 hours. GSK-2126458, a Phosphoinositide 3-kinase inhibitor, demonstrated similarity at 20 minutes and 6 hours. The MEK/ERK pathway inhibitor PD-0325901 exhibited similarity at 20 minutes and 2 hours. Protein Kinase C inhibitor Ro 21-8220 mesylate demonstrated a similar effect at 60 minutes-treatment of FX06.

**Figure 11:**
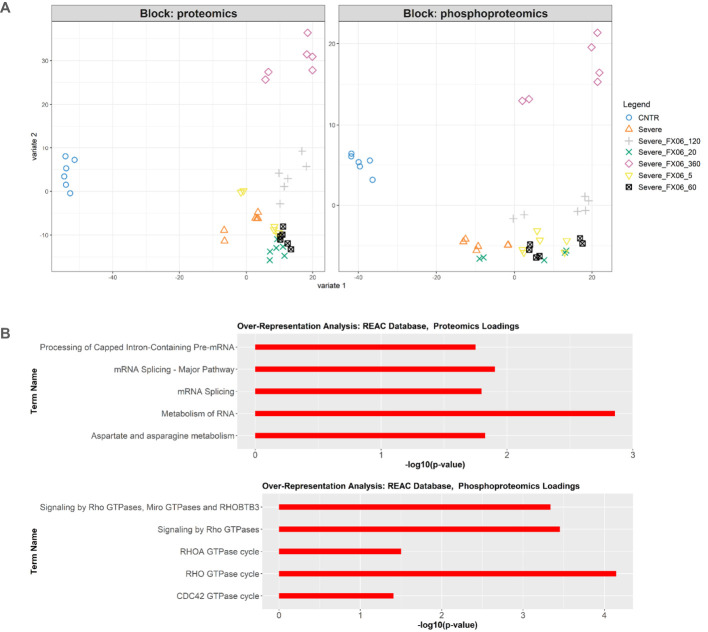
**(A)** DIABLO sample plot based on sample scores on first (Variable 1) and second (Variable 2) components from proteomics and phosphoproteomics data. **(B)** Overrepresentation analysis of top 100 proteomics and phosphoproteomics loadings of component 2 based on Reactome database. Only statistically significant pathways are selected (p-value < 0.05). The length of bars represents -log10 (p-value).

Integration of proteomics and phosphoproteomics data using the DIABLO method also reveals inactivation of RhoGTPase cycle signaling by FX06. The integrated analysis shows distinct profiles between untreated ECs and treated ECs (**Figure 11A**). FX06-treated ECs gradually become distinct from cells treated with the severe cytokine cocktail over time. The difference between the two groups can be observed in phosphorylation changes in RhoGTPase (**Figure 11B**). The severe cytokine cocktail causes an increase in phosphorylation of proteins involved in the RhoGTPase cycle, and FX06 treatment at all time points downregulates the RhoGTPase cycle. A similar effect was demonstrated by proteomics data where FX06 reduces the protein abundance associated with Rho signaling in the differential expression analysis (**Figure 9C**). For instance, FX06 treatment for 60 and 120 minutes in the presence of the severe cytokine cocktail decreases PTPRF abundance. FX06 treatment for 120 and 360 minutes decreases CCNA2 abundance.

### 3.10 FX06 may restore the integrity of the vascular wall by interfering with the RhoA-ROCK-1 signal transduction pathway

**Figure 12:**
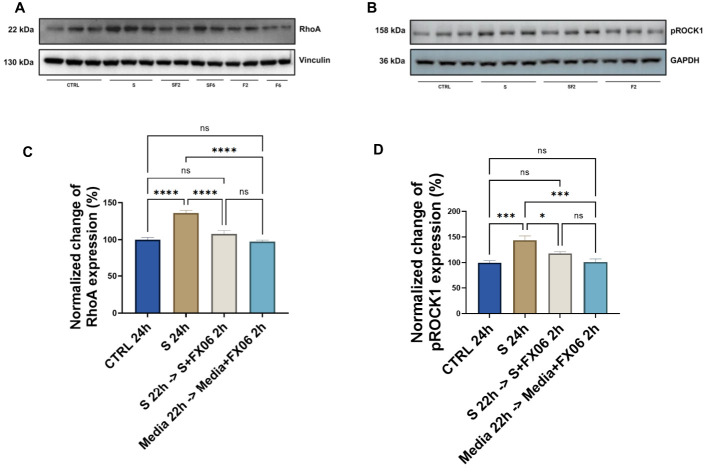
The effects of the severe cytokine cocktail alone, and the severe cytokine cocktail in the presence of FX06 on the total expression of RhoA and phospho-ROCK1 in HULEC-5a **(C, D)** Protein lysates were collected for Western blots to measure RhoA and phospho-ROCK1 with an anti-RhoA antibody and anti-Phospho-ROCK1 (Thr455, Ser456). Anti-vinculin and anti-GAPDH as a housekeeping marker were used to normalise data. A representative blot of three independent experiments is shown above. **(A, B)** Western bands were quantified via ImageJ. Quantification data from three and six independent experiments regarding RhoA and phospho-ROCK1 expression is shown in the graph. 2 h-treatment of FX06 decreased the RhoA expression and phospho-ROCK1 that was enhanced by the severe cytokine cocktail. One-way ANOVA, ns: not significant, *P<0.05, ***P<0.001, ****P<0.0001.

It has been mentioned previously that FX06 inhibits the RhoA activation via Fyn, to maintain the EC barrier (27). As proteomics and phosphoproteomics data detected that FX06 treatment normalized the cytokine-induced RhoA overexpression, their effect on the modulation of the RhoA signaling pathways was further verified by Western Blot. ECs were treated with medium and the severe cytokine cocktail for 24 h, in the absence of presence of FX06. RhoA abundance increased after the severe cytokine cocktail treatment, while FX06 reversed this change returning RhoA to the control level (as shown in **Figures 12A, C**). We further investigated ROCK1, a downstream effector of RhoA activated upon RhoA phosphorylation involved in decreasing endothelial integrity (49). Similarly to RhoA expression, FX06 treatment reversed the cytokine-induced overactivation of ROCK1 to the control level (**Figures 12B, D**). The full scan of original gels were shown in **Supplementary Figure S7**. The results indicated that FX06 restores the normal barrier function of endothelium through RhoA-pROCK1 pathway (50).

## 4 Discussion

Endothelial dysfunction plays a pivotal role in the pathogenesis of various inflammatory diseases, including COVID-19 (13). Addressing endothelial dysfunction could be a potential adjuvant therapy for preventing the progression of COVID-19 and other conditions that involve endothelial dysfunction, for example ARDS, to severe disease.

This study aims to mimic the inflammatory status of SARS-CoV-2 infection by treating ECs with COVID-19-triggered cytokines and to investigate whether FX06 can protect from endothelial dysfunction. In the proteomics study, the severe cytokine cocktail promotes inflammatory reactions, marked by an increase in the abundance of proteins involved in pro-inflammatory pathways. For instance, enhanced expression levels of GBP1, an essential protein for innate immunity and a promoter of inflammasome activation, are associated with the activation of neutrophils and the NOD receptor signaling pathway (51). NFKB2, a subunit of the transcription factor complex nuclear factor-kappa-B, enhanced by the severe cytokine cocktail, is involved in the expression of various proinflammatory markers, including chemokines, cytokines, and adhesion molecules (52). RELB, WARS1, HLA-B, IFIT2 and IL15RA are also involved in inflammation (53–57). ICAM1, an adhesion receptor involved in regulating leukocyte recruitment to the sites of inflammation (58), is also increased in response to the severe cytokine cocktail stimulation.

The anti-inflammatory effects of FX06 have been demonstrated by its ability to prevent leukocyte migration induced by the fibrin fragment N-terminal disulfide knot (NDSK)-II, through ECs (59). In the present study, FX06 decreases the TEM of PBMCs through ECs treated with COVID-19-triggered cytokines suggesting that FX06 can reduce vascular leakage caused by COVID-19-triggered cytokines. Since SARS-CoV-2 infects ECs through the S1 protein (60), we established a model of ECs infection with S1, along with exposure to COVID-19-triggered cytokines simultaneously which confirmed the ability of FX06 to decreased the immune cell infiltration. FX06 cannot protect ECs against apoptotic or necrotic effects of COVID-19-triggered cytokines, suggesting that the restoring effect of FX06 must be through other means. FX06 at 2 h treatment decreases the severe cytokine cocktail-induced enhancement in TNFRSF17 abundance. TNFRSF is associated with immune infiltration (61). Two hours-FX06-treatment also decreases CD47 abundance. The engagement of CD47 results in an increase in the permeability of ECs, the blockade of CD47 decreases T cell transmigration (62). FX06 enhances the activity of several pathways important for endothelial barrier function. The increased ERBB signaling can as well contribute to the protection against capillary leakage as downregulation of ERBB3 has been shown to significantly increase blood-brain barrier permeability (63). Both tight and gap junctions are vital for endothelial barriers. The stimulation of gonadotropin-releasing hormone signaling can significantly reduce the expressions of VEGF and its receptor (25, 64), which could be a protective mechanism of FX06 on ECs as VEGF can uncouple endothelial cell–cell junctions (65).

In the present study, the severe cytokine cocktail enhances angiogenesis compared to untreated groups, confirming that proinflammatory cytokines during inflammation promote angiogenesis (66). ECs undergo morphological changes, such as EC elongation, during the formation of vascular networks (45). The severe cytokine cocktail causes morphological changes in ECs by increasing cell size and length. The new vessels formed in inflammation facilitate the continuing recruitment of inflammatory cells (67). The presence of FX06 in severe cytokine cocktails can reduce angiogenic activity, indicating that FX06 can inhibit the excessive vascular vessel formation, and potentially reduce the angiogenesis-induced inflammation. At first glance, it looks peculiar that an endothelial-protective drug such as FX06 can revert angiogenesis. But as soon as the angiogenic signal is associated with inflammation or any other kind of danger signal (e.g. tumor angiogenesis), endothelial-protective drugs tend to normalize the vasculature, as evidenced in previously published reports on a different endothelium protecting compound called Defibrotide (68, 69).

RhoGTPase is of particular interest as the severe cytokine cocktail increases the phosphorylation of proteins related to RhoGTPase cycles, and FX06 decreases it. Additionally, FX06 treatment in the presence of the severe cytokine cocktail suppresses the PTPRT and CCNA2 abundance associated with Rho signaling (70, 71). It has been confirmed in our study that FX06 reduces the upregulated RhoA expression induced by COVID-19-triggered cytokines. Rho-associated kinases ROCK is the downstream protein of the small GTPases RhoA (72). The severe cytokine cocktail increases the expression of phospho-ROCK-1, an activated form of ROCK-1, which contributes to enhanced angiogenic processes (73). Addition of FX06 to the severe cytokine cocktail decreases enhanced expression of phospho-ROCK-1, decreasing angiogenesis accordingly. Taken together, FX06 works as a RhoA-ROCK pathway inhibitor that can reduce angiogenesis. Changes in RhoA expression could also potentially explain the reduced TEM of PBMCs. It has been revealed that FX06 antagonizes thrombin-induced RhoA activation by associating Fyn with p190RhoGAP, to reduce vascular leakage (16, 45). Mikelis et al. have investigated the role of RhoA in endothelial permeability in both *in vitro* and *in vivo* studies and found that RhoA inhibition prevents endothelial leakage (74). Angiogenesis is accompanied by increased permeability allowing new capillary sprout (73). Deceased RhoA together with decreased activity of RhoA-ROCK-1 pathway-induced angiogenesis by FX06 both contribute to the normalized integrity of endothelium and the normalization of vasculature.

Reorganization of the vascular barrier potentially compensates for the apparent cell loss. As VE-Cadherin is generally vital for maintaining the integrity of the EC monolayer, investigating VE-Cadherin is of great importance (75). In our study, COVID-19-triggered cytokines disrupt the continuous surface expression of VE-Cadherin on ECs, whereas FX06 can partially restore this. Our observation is in line with previously published literature. Gröger et al. reported that FX06 promotes the continuity of VE-Cadherin lining in ECs treated with thrombin (16). Similarly, Wu et al. found that FX06 prevents the formation of gaps of VE-Cadherin between ECs, thus decreasing vascular permeability (15). This evidence reveals that FX06 protects the integrity of the vascular lining by adjusting VE-Cadherin distribution on ECs. It has also been reported that closing gaps between ECs by FX06 can also explain the anti-inflammatory role of FX06 (76).

CD138, an important component of endothelial glycocalyx, works as an endothelial gatekeeper to maintain vascular permeability (77). Wu et al. reported that fibrinogen interacts with CD138 and activates PAK1/Cofilin signaling pathways, to maintain endothelial integrity (78, 79). SDC1-knockout mice models had sustained neutrophilic inflammation while inflammation in WT mice was resolved (80). It has been observed that TNF-α and IL-1β induce the loss of membrane CD138 in epithelial cells, IL-6 does not cause any changes (81). Different cytokines or chemokines might alter CD138 expression in different ways. However, in the present study, we observed that COVID-19-triggered cytokines induce over-expression of CD138 on the surface of ECs. Marshall et al. revealed that IL-8, one type of chemokine in our severe cytokine cocktail, forms a complex with CD138 and generates a chemokine gradient as a prerequisite for neutrophil transendothelial migration (82). Elevated expression of CD138 caused by severe cytokine cocktails potentially facilitates more TEM. The serum level of CD138, a component of the glycocalyx of ECs, is associated with the degradation of the endothelium (42). Serum CD138, due to CD138 shedding from the endothelial surface, is associated with mortality in COVID-19 patients (42, 43). CD138 was significantly higher in serum samples of severe COVID-19 patients than in healthy controls (42, 43). CD138 is closely related to disease condition changes in COVID-19 patients and is considered a prognostic marker for COVID-19 patients (83, 84). However, in the *in vitro* study, cytokines in the presence of FX06 cause higher levels of shed CD138 as compared to severe cytokines alone. The enhanced shedding of IL-8 and CD138 complex inhibits neutrophil transendothelial migration (85). Hayashida et al. also revealed CD138 shedding facilitates the resolution of neutrophilic inflammation by the clearance of chemokine gradient, thus halting neutrophil infiltration (80). FX06 treatment reverses the elevated surface level of CD138 back to normal, and more shedding of CD138 assists in the resolution of inflammation. There was no correlation between serum levels of CD138 and COVID-19 disease severity (42). Whether CD138 can be a therapeutic biomarker or monitor of disease progression in clinical practice still needs further investigation.

Gröger et al. demonstrated that FX06 prevents the stress fiber formation caused by thrombin (16). In our study, we found that COVID-19-triggered cytokines induce higher intensity of F-actin fiber formation, and the length of actin fibers is increased together with elongated cell length, whereas FX06 shortens the length of stress fibers but does not change the expression level of F-actin fibers. FX06 can potentially relieve stress of ECs caused by COVID-19-triggered cytokines. Mfuzz clustering analysis based on phosphoproteomics also showed that FX06 can reverse changes on cytoskeletal changes caused by the severe cytokine cocktail.

A proinflammatory status can also be caused by cellular cytotoxicity of either autologous or allogeneic CD8^+^ T cells. We observed that COVID-19-triggered cytokines significantly enhance the allogenicity of human pulmonary ECs towards allogeneic CD8^+^ CTL which can be reversed by FX06. This comprises another anti-inflammatory function of the drug that belongs to the endothelial-stabilizing nature of FX06, just like it had been observed with a similar drug called Defibrotide (86).

Overall, FX06 shows great potential in reducing vascular leakage to protect the endothelium against the proinflammatory effect of COVID-19-triggered cytokines. The protective MOA of FX06 is not due to anti-apoptotic or anti-necrotic effects. However, FX06 shows great potential for reducing the inflammation by tightening up junctions between ECs, by decreasing angiogenic activity and limiting cell contraction. The effects of FX06 for the different treatment period of time in RhoA-ROCK signal transduction pathway and VEGF related signaling pathway might be promising for future research to even better understand its mechanism of action. In clinical studies, it has been reported that FX06 is safe to be applied in COVID-19 patients, and previously has been proven safe in patients with myocardial infarction (87, 88). FX06 improved the oxygenation ability and lung function in patients diagnosed with SARS-CoV-2-induced ARDS in a small group of six patients, which suggests FX06 protects pulmonary vascular barriers (19). In contrast, in a larger-sized study in critically ill patients, FX06 did not reduce pulmonary vascular leakage in patients with SARS-CoV-2-induced ARDS (87), suggesting that FX06 may have to be applied at earlier time points, less severe cases and/or different dosage regimen. Although acute COVID-19 has now reasonably been managed by appropriate vaccination against SARS-CoV-2, FX06 might be a promising treatment measure in further diseases associated with capillary leakage. As host- directed therapy, FX06 could be beneficial in currently known viral diseases with compromised endothelial function like Lassa fever or severe Dengue but also for treating future - so far unknown - pathogens affecting the endothelium.

## Data Availability

The datasets presented in this study can be found in online repositories. The names of the repository/repositories and accession number(s) can be found in the article/**Supplementary Material**.
