# Supplementary material for: The fibrin-derived peptide FX06 protects human pulmonary endothelial cells against the COVID-19-triggered cytokine storm
Source: Front Immunol. 2025 Jun 19;16:1591860. doi: 10.3389/fimmu.2025.1591860 (PMC12225545; doi:10.3389/fimmu.2025.1591860)
Supplement: Supplementary file 1 [file SupplementaryFile1.docx]

Table S3

| **Cell Group** | **Rank** | **Score** | **Perturbation** |
| --- | --- | --- | --- |
| FX06-5 min vs Severe | 1 | 0.0952 | CGP 71683 hydrochloride |
| FX06-5 min vs Severe | 2 | 0.0952 | Hinokitiol |
| FX06-5 min vs Severe | 3 | 0.0952 | Hinokitiol |
| FX06-5 min vs Severe | 4 | 0.0952 | SIB 1893 |
| FX06-5 min vs Severe | 5 | 0.0952 | wortmannin |
| FX06-5 min vs Severe | 6 | 0.0952 | Simvastatin |
| FX06-5 min vs Severe | 7 | 0.0952 | BMS-536924 - Inhibitor of Insulin Growth Factor-IR |
| FX06-5 min vs Severe | 8 | 0.0952 | MW-ras12 |
| FX06-5 min vs Severe | 9 | 0.0952 | TG101348 |
| FX06-5 min vs Severe | 10 | 0.0952 | 528116.cdx |
| FX06-20 min vs Severe | 1 | 0.1111 | BMS-536924 - Inhibitor of Insulin Growth Factor-IR |
| FX06-20 min vs Severe | 2 | 0.1111 | BRD-U25771771 |
| FX06-20 min vs Severe | 3 | 0.1111 | trichostatin A |
| FX06-20 min vs Severe | 4 | 0.0926 | AS605240 - Phosphatidylinositol-3-Kinase Inhibitor |
| FX06-20 min vs Severe | 5 | 0.0926 | L-sulforaphane |
| FX06-20 min vs Severe | 6 | 0.0926 | BRD-K08307026 |
| FX06-20 min vs Severe | 7 | 0.0926 | vorinostat |
| FX06-20 min vs Severe | 8 | 0.0926 | vorinostat |
| FX06-20 min vs Severe | 9 | 0.0926 | GSK-2126458 - Phosphatidylinositol-3-Kinase Inhibitor |
| FX06-20 min vs Severe | 10 | 0.0926 | GSK-2126458 - Phosphatidylinositol-3-Kinase Inhibitor |
| FX06-60 min vs Severe | 1 | 0.0874 | BRD-K70161581 |
| FX06-60 min vs Severe | 2 | 0.0874 | BRD-U25771771 |
| FX06-60 min vs Severe | 3 | 0.0777 | BMS-536924 - Inhibitor of Insulin Growth Factor-IR |
| FX06-60 min vs Severe | 4 | 0.0777 | S1122 |
| FX06-60 min vs Severe | 5 | 0.0777 | BRD-K38615104 |
| FX06-60 min vs Severe | 6 | 0.0777 | BRD-U00779237 |
| FX06-60 min vs Severe | 7 | 0.068 | cucurbitacin I |
| FX06-60 min vs Severe | 8 | 0.068 | F3055 |
| FX06-60 min vs Severe | 9 | 0.068 | BRD-K19220233 |
| FX06-60 min vs Severe | 10 | 0.068 | BRD-K82823804 |
| FX06-120 min vs Severe | 1 | 0.0741 | BMS-536924 - Inhibitor of Insulin Growth Factor-IR |
| FX06-120 min vs Severe | 2 | 0.0741 | vemurafenib |
| FX06-120 min vs Severe | 3 | 0.0741 | foretinib |
| FX06-120 min vs Severe | 4 | 0.0648 | KU 0060648 trihydrochloride |
| FX06-120 min vs Severe | 5 | 0.0648 | HY-50940 |
| FX06-120 min vs Severe | 6 | 0.0648 | 5122-2566 |
| FX06-120 min vs Severe | 7 | 0.0648 | S1230 |
| FX06-120 min vs Severe | 8 | 0.0648 | BRD-K38615104 |
| FX06-120 min vs Severe | 9 | 0.0648 | BRD-A73909368 |
| FX06-120 min vs Severe | 10 | 0.0648 | CGP-60474 |
| FX06-360 min vs Severe | 1 | 0.0407 | GSK-2126458 - Phosphatidylinositol-3-Kinase Inhibitor |
| FX06-360 min vs Severe | 2 | 0.037 | BRD-K26664453 |
| FX06-360 min vs Severe | 3 | 0.037 | PROSTAGLANDIN A1 |
| FX06-360 min vs Severe | 4 | 0.037 | HY-10005 |
| FX06-360 min vs Severe | 5 | 0.037 | EI-232 |
| FX06-360 min vs Severe | 6 | 0.0333 | atorvastatin |
| FX06-360 min vs Severe | 7 | 0.0333 | PP-110 |
| FX06-360 min vs Severe | 8 | 0.0333 | BRD-K12184916 |
| FX06-360 min vs Severe | 9 | 0.0333 | PI 103 hydrochloride |
| FX06-360 min vs Severe | 10 | 0.0333 | AZD8055 |
