# Supplementary material for: The fibrin-derived peptide FX06 protects human pulmonary endothelial cells against the COVID-19-triggered cytokine storm
Source: Front Immunol. 2025 Jun 19;16:1591860. doi: 10.3389/fimmu.2025.1591860 (PMC12225545; doi:10.3389/fimmu.2025.1591860)
Supplement: Supplementary file 2 [file SupplementaryFile2.pptx]

## Slide 1
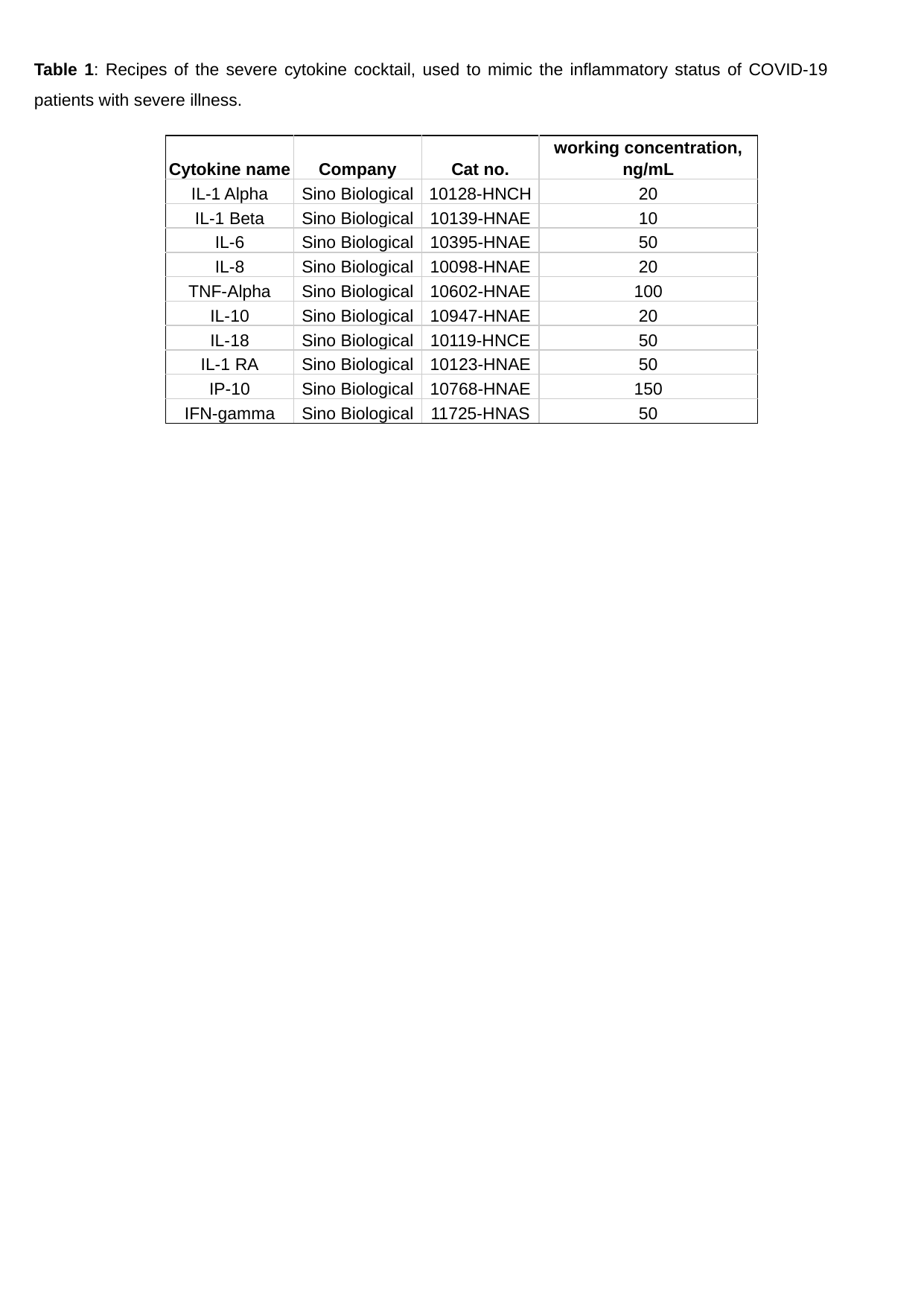

Table 1: Recipes of the severe cytokine cocktail, used to mimic the inflammatory status of COVID-19 patients with severe illness.
| Cytokine name | Company | Cat no. | working concentration, ng/mL |
| --- | --- | --- | --- |
| IL-1 Alpha | Sino Biological | 10128-HNCH | 20 |
| IL-1 Beta | Sino Biological | 10139-HNAE | 10 |
| IL-6 | Sino Biological | 10395-HNAE | 50 |
| IL-8 | Sino Biological | 10098-HNAE | 20 |
| TNF-Alpha | Sino Biological | 10602-HNAE | 100 |
| IL-10 | Sino Biological | 10947-HNAE | 20 |
| IL-18 | Sino Biological | 10119-HNCE | 50 |
| IL-1 RA | Sino Biological | 10123-HNAE | 50 |
| IP-10 | Sino Biological | 10768-HNAE | 150 |
| IFN-gamma | Sino Biological | 11725-HNAS | 50 |

## Slide 2
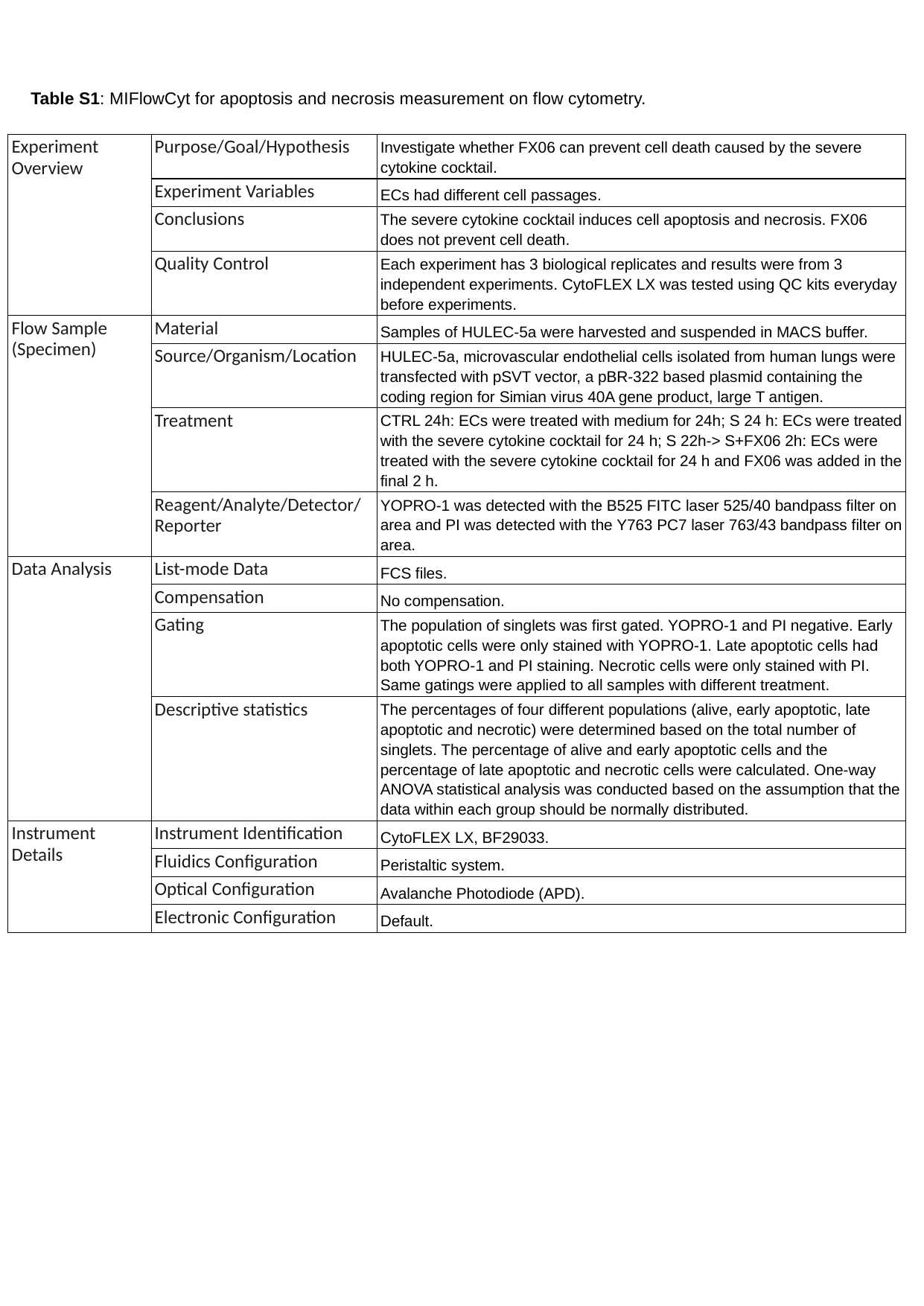

Table S1: MIFlowCyt for apoptosis and necrosis measurement on flow cytometry.
| Experiment Overview | Purpose/Goal/Hypothesis | Investigate whether FX06 can prevent cell death caused by the severe cytokine cocktail. |
| --- | --- | --- |
| | Experiment Variables | ECs had different cell passages. |
| | Conclusions | The severe cytokine cocktail induces cell apoptosis and necrosis. FX06 does not prevent cell death. |
| | Quality Control | Each experiment has 3 biological replicates and results were from 3 independent experiments. CytoFLEX LX was tested using QC kits everyday before experiments. |
| Flow Sample (Specimen) | Material | Samples of HULEC-5a were harvested and suspended in MACS buffer. |
| | Source/Organism/Location | HULEC-5a, microvascular endothelial cells isolated from human lungs were transfected with pSVT vector, a pBR-322 based plasmid containing the coding region for Simian virus 40A gene product, large T antigen. |
| | Treatment | CTRL 24h: ECs were treated with medium for 24h; S 24 h: ECs were treated with the severe cytokine cocktail for 24 h; S 22h-> S+FX06 2h: ECs were treated with the severe cytokine cocktail for 24 h and FX06 was added in the final 2 h. |
| | Reagent/Analyte/Detector/Reporter | YOPRO-1 was detected with the B525 FITC laser 525/40 bandpass filter on area and PI was detected with the Y763 PC7 laser 763/43 bandpass filter on area. |
| Data Analysis | List-mode Data | FCS files. |
| | Compensation | No compensation. |
| | Gating | The population of singlets was first gated. YOPRO-1 and PI negative. Early apoptotic cells were only stained with YOPRO-1. Late apoptotic cells had both YOPRO-1 and PI staining. Necrotic cells were only stained with PI. Same gatings were applied to all samples with different treatment. |
| | Descriptive statistics | The percentages of four different populations (alive, early apoptotic, late apoptotic and necrotic) were determined based on the total number of singlets. The percentage of alive and early apoptotic cells and the percentage of late apoptotic and necrotic cells were calculated. One-way ANOVA statistical analysis was conducted based on the assumption that the data within each group should be normally distributed. |
| Instrument Details | Instrument Identification | CytoFLEX LX, BF29033. |
| | Fluidics Configuration | Peristaltic system. |
| | Optical Configuration | Avalanche Photodiode (APD). |
| | Electronic Configuration | Default. |

## Slide 3
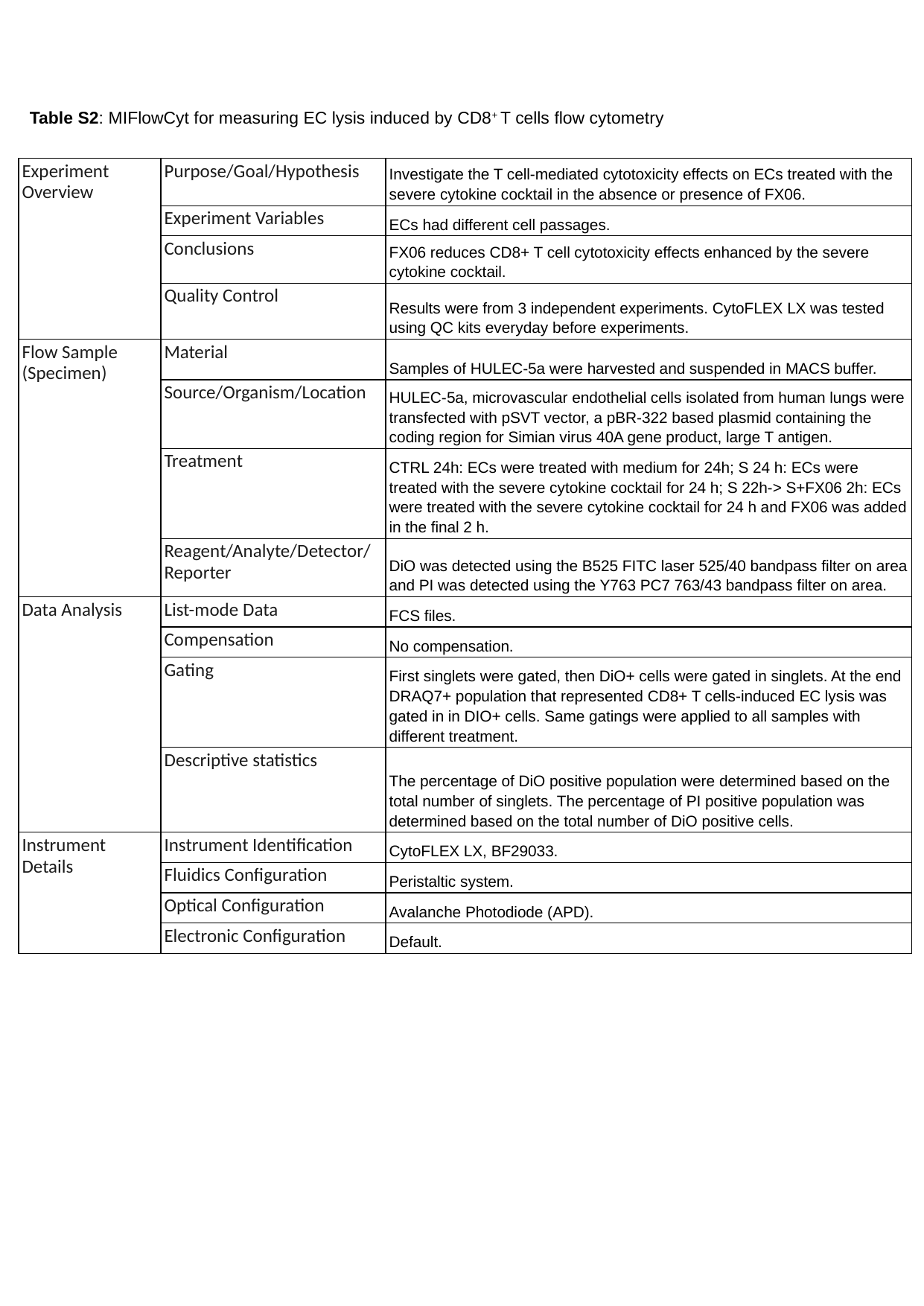

Table S2: MIFlowCyt for measuring EC lysis induced by CD8+ T cells flow cytometry
| Experiment Overview | Purpose/Goal/Hypothesis | Investigate the T cell-mediated cytotoxicity effects on ECs treated with the severe cytokine cocktail in the absence or presence of FX06. |
| --- | --- | --- |
| | Experiment Variables | ECs had different cell passages. |
| | Conclusions | FX06 reduces CD8+ T cell cytotoxicity effects enhanced by the severe cytokine cocktail. |
| | Quality Control | Results were from 3 independent experiments. CytoFLEX LX was tested using QC kits everyday before experiments. |
| Flow Sample (Specimen) | Material | Samples of HULEC-5a were harvested and suspended in MACS buffer. |
| | Source/Organism/Location | HULEC-5a, microvascular endothelial cells isolated from human lungs were transfected with pSVT vector, a pBR-322 based plasmid containing the coding region for Simian virus 40A gene product, large T antigen. |
| | Treatment | CTRL 24h: ECs were treated with medium for 24h; S 24 h: ECs were treated with the severe cytokine cocktail for 24 h; S 22h-> S+FX06 2h: ECs were treated with the severe cytokine cocktail for 24 h and FX06 was added in the final 2 h. |
| | Reagent/Analyte/Detector/Reporter | DiO was detected using the B525 FITC laser 525/40 bandpass filter on area and PI was detected using the Y763 PC7 763/43 bandpass filter on area. |
| Data Analysis | List-mode Data | FCS files. |
| | Compensation | No compensation. |
| | Gating | First singlets were gated, then DiO+ cells were gated in singlets. At the end DRAQ7+ population that represented CD8+ T cells-induced EC lysis was gated in in DIO+ cells. Same gatings were applied to all samples with different treatment. |
| | Descriptive statistics | The percentage of DiO positive population were determined based on the total number of singlets. The percentage of PI positive population was determined based on the total number of DiO positive cells. |
| Instrument Details | Instrument Identification | CytoFLEX LX, BF29033. |
| | Fluidics Configuration | Peristaltic system. |
| | Optical Configuration | Avalanche Photodiode (APD). |
| | Electronic Configuration | Default. |
