# Supplementary figures and images for: The fibrin-derived peptide FX06 protects human pulmonary endothelial cells against the COVID-19-triggered cytokine storm
Source: Front Immunol. 2025 Jun 19;16:1591860. doi: 10.3389/fimmu.2025.1591860 (PMC12225545; doi:10.3389/fimmu.2025.1591860)

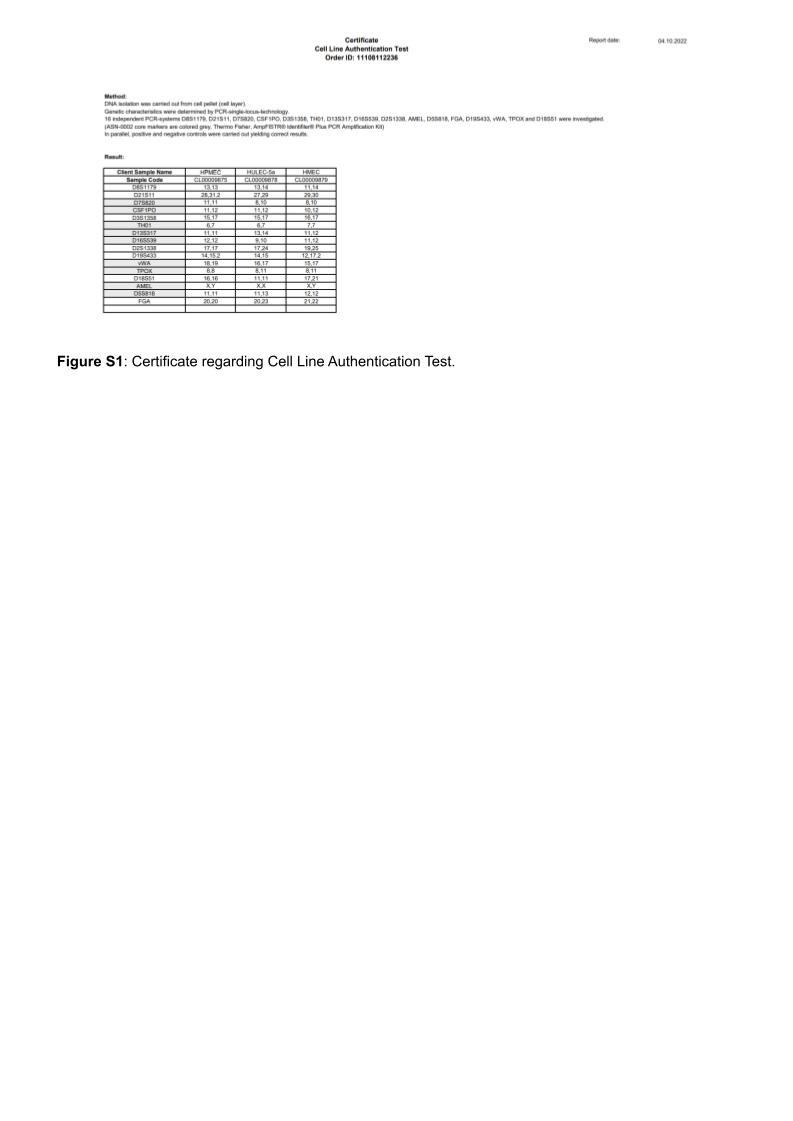

Supplement: Supplementary file 3 [file Image1.jpeg]

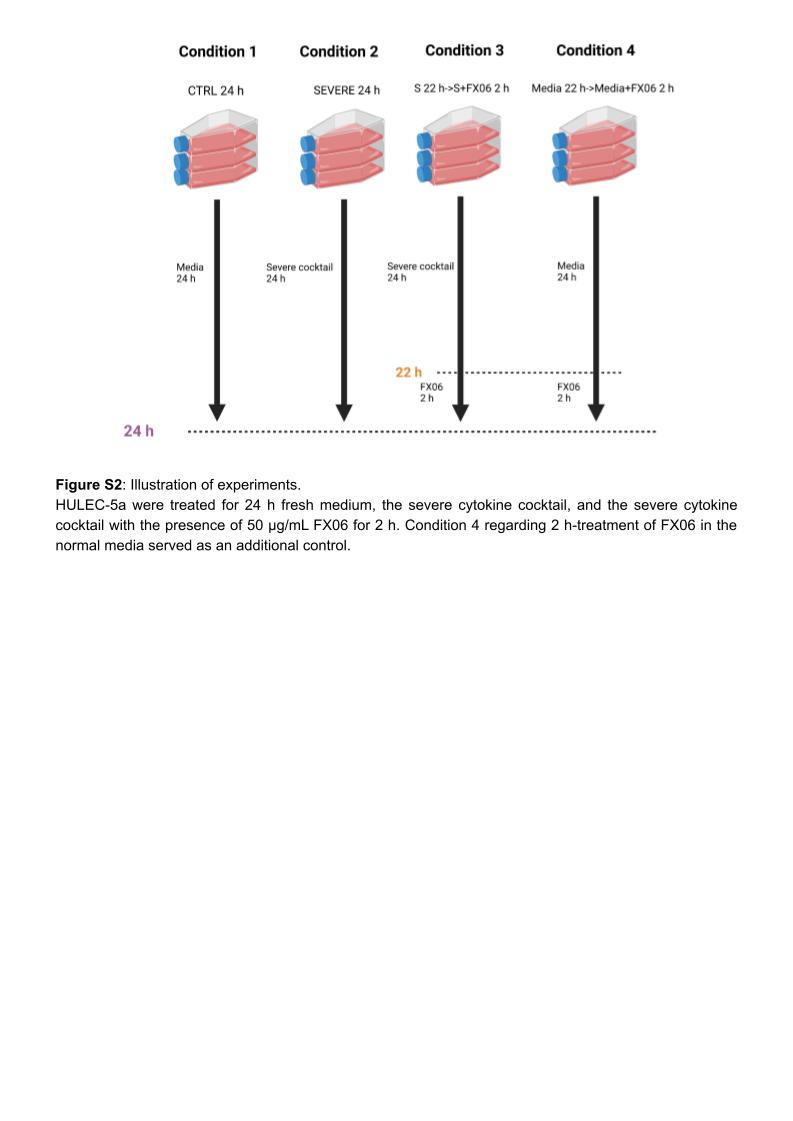

Supplement: Supplementary file 4 [file Image2.jpeg]

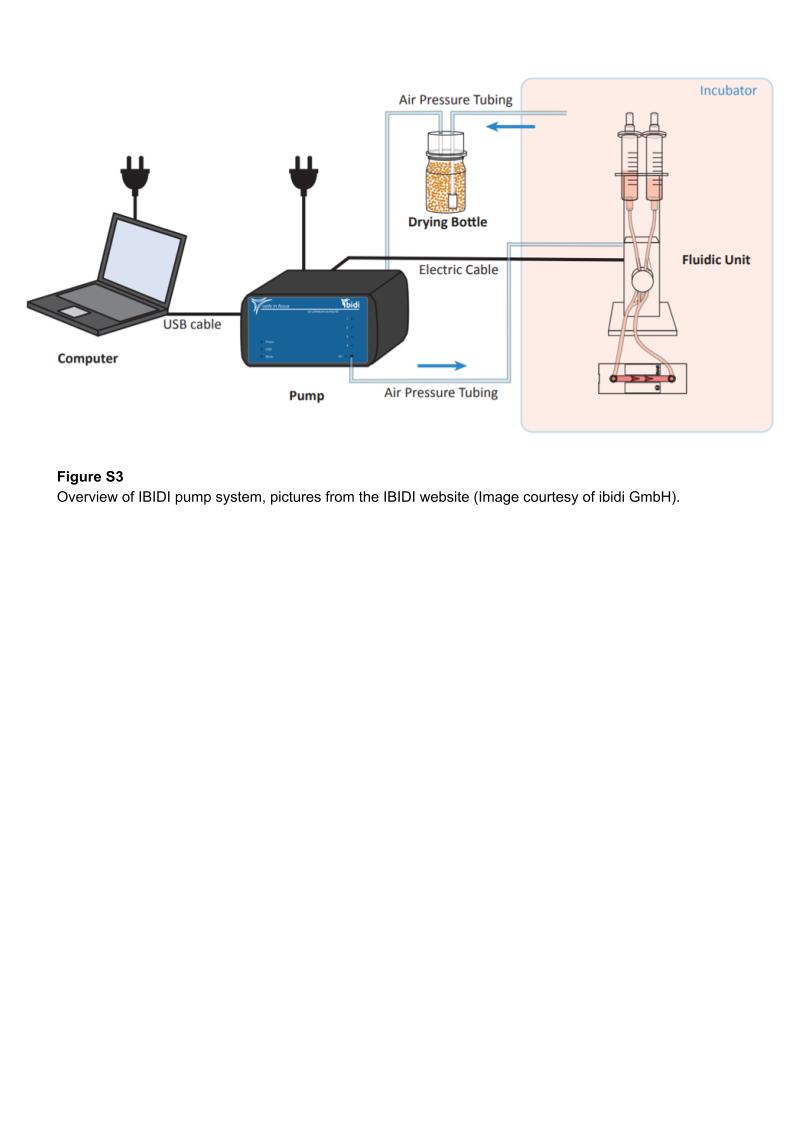

Supplement: Supplementary file 5 [file Image3.jpeg]

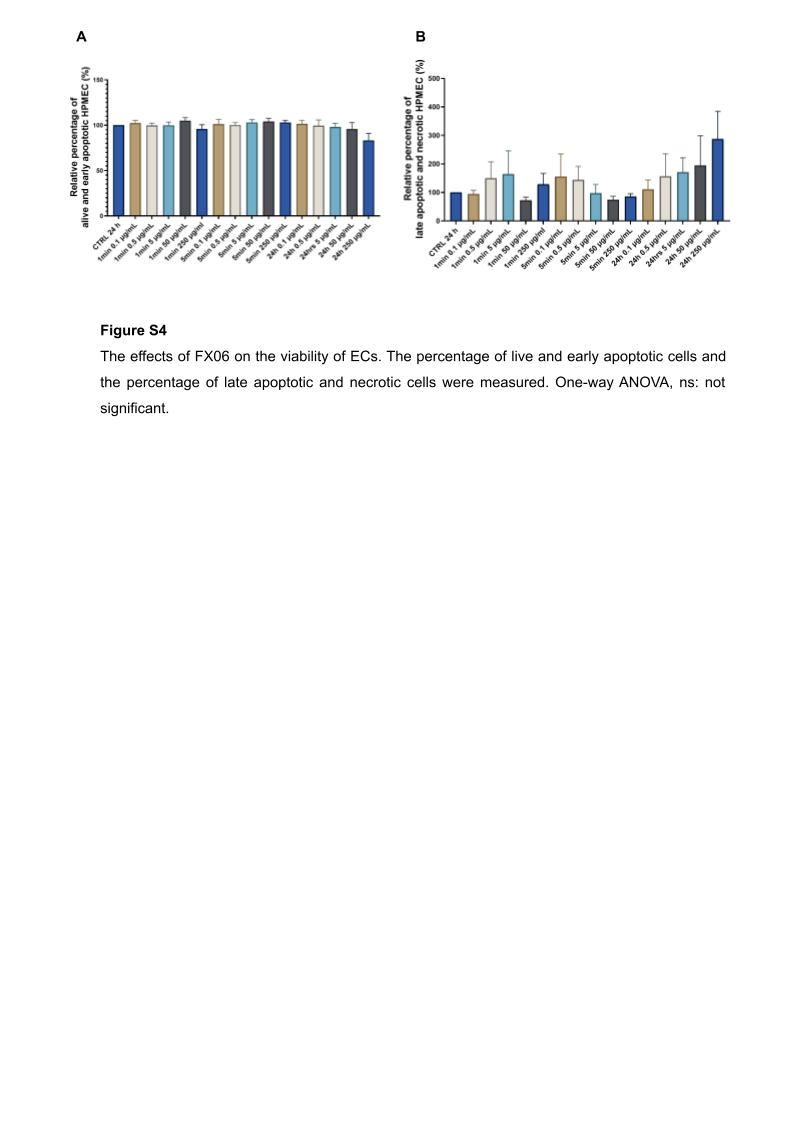

Supplement: Supplementary file 6 [file Image4.jpeg]

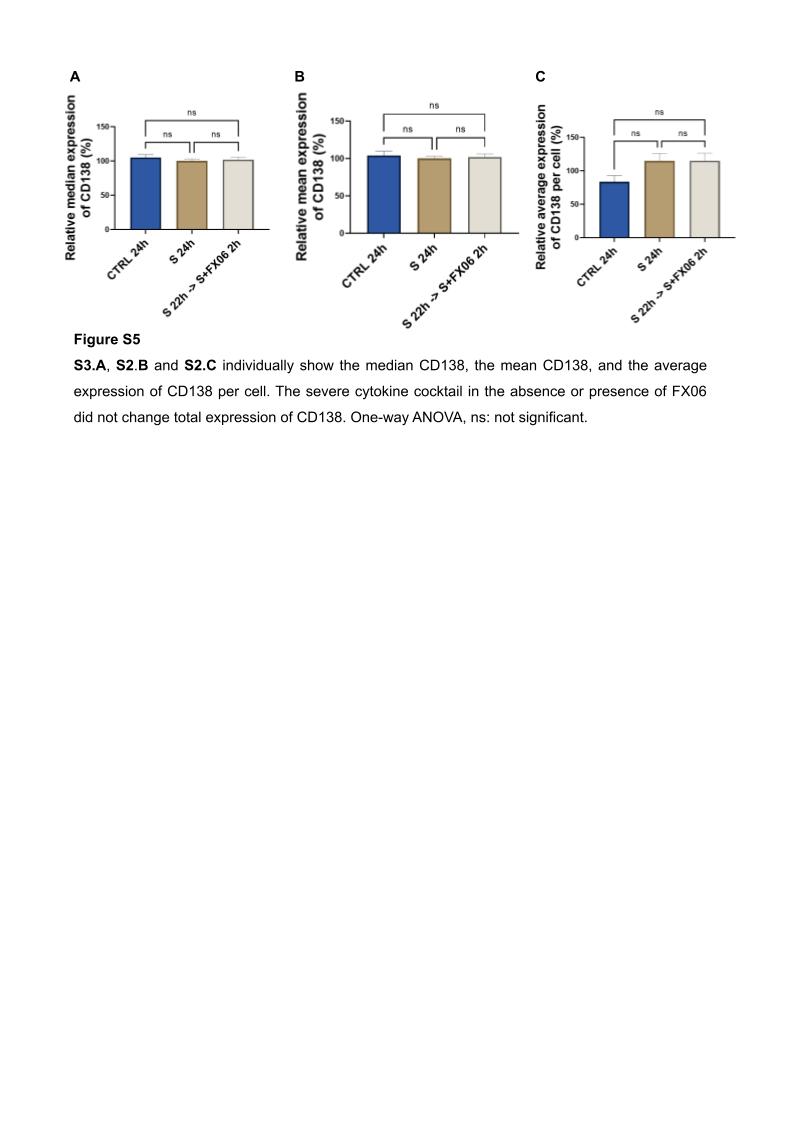

Supplement: Supplementary file 7 [file Image5.jpeg]

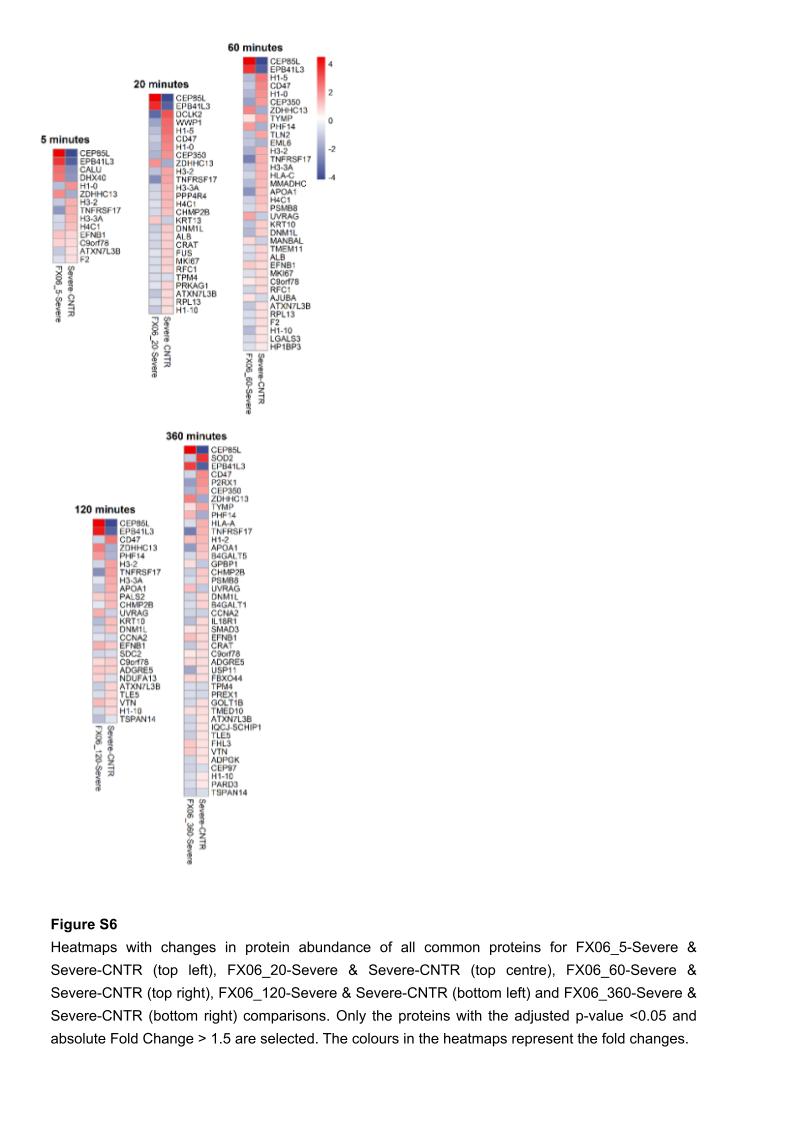

Supplement: Supplementary file 8 [file Image6.jpeg]

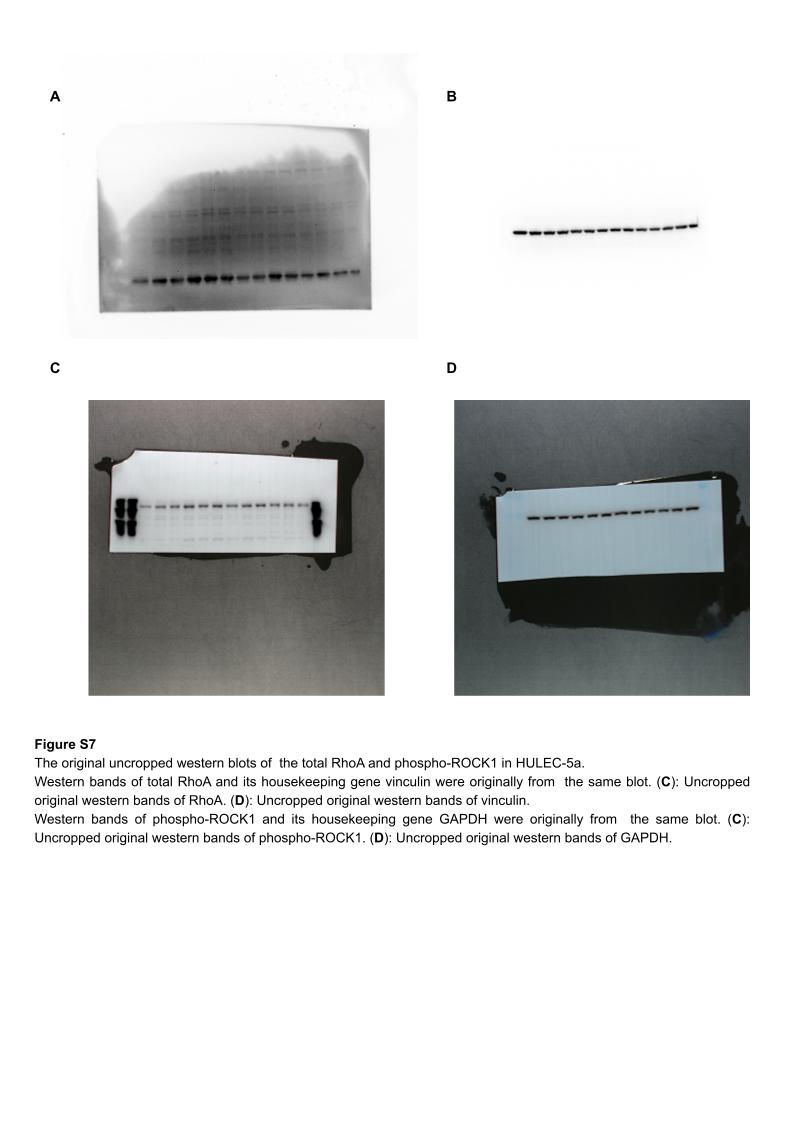

Supplement: Supplementary file 9 [file Image7.jpeg]

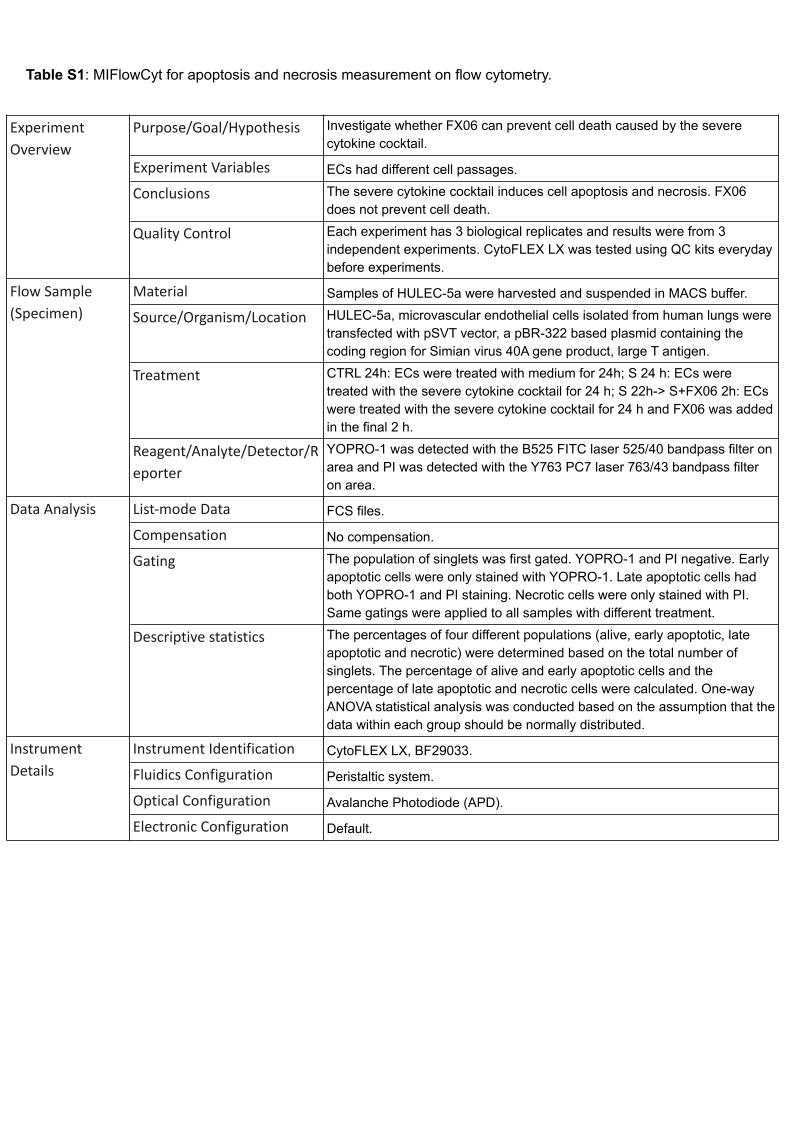

Supplement: Supplementary file 10 [file Image8.jpeg]

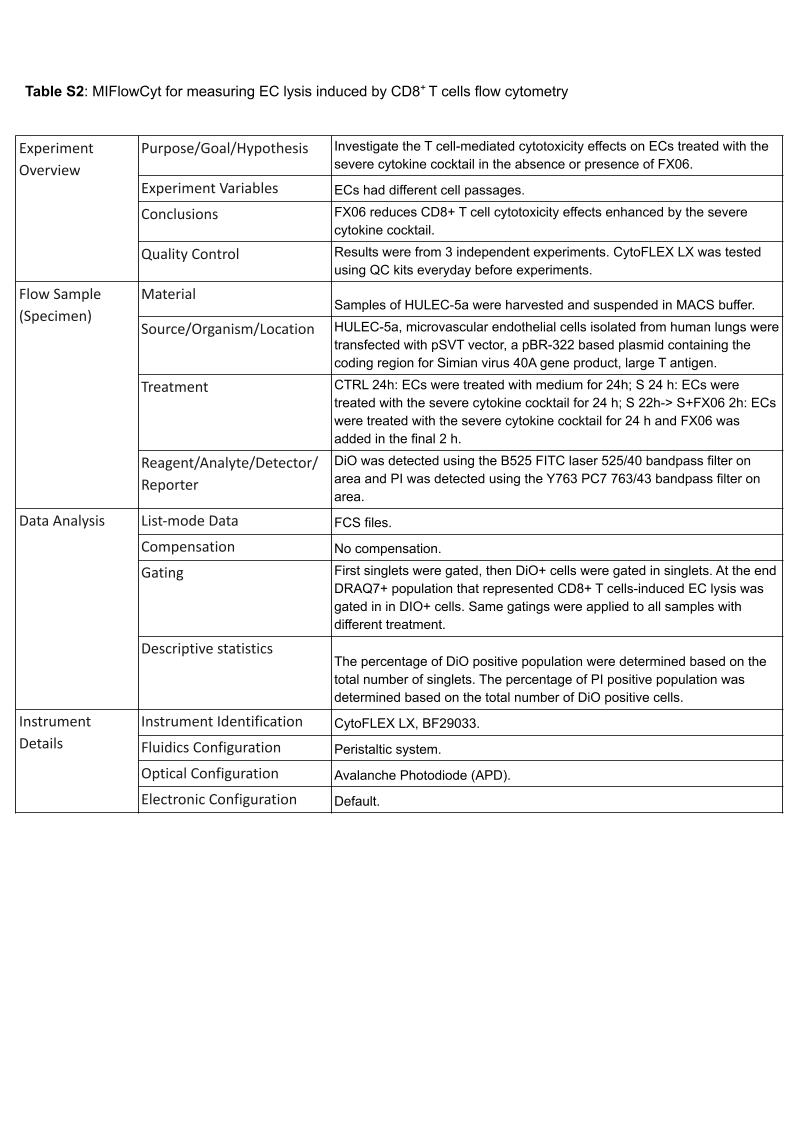

Supplement: Supplementary file 11 [file Image9.jpeg]

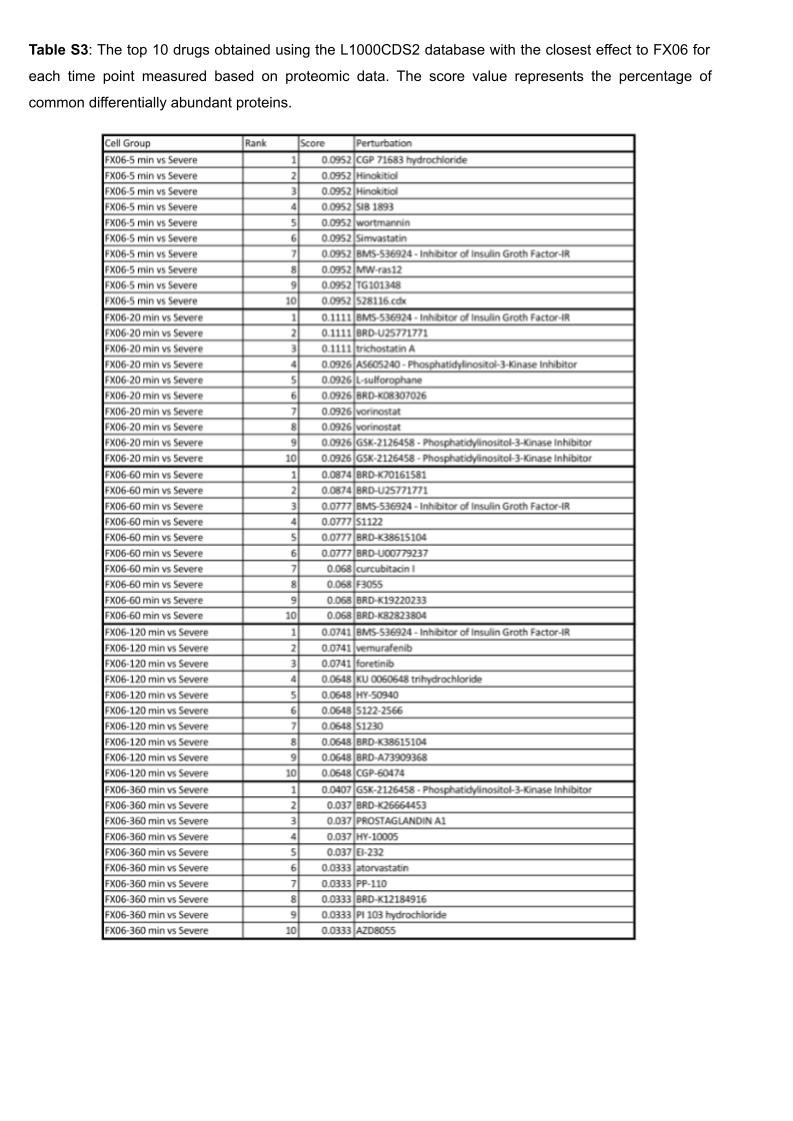

Supplement: Supplementary file 12 [file Image10.jpeg]
